# ChiLin: a comprehensive ChIP-seq and DNase-seq quality control and analysis pipeline

**DOI:** 10.1186/s12859-016-1274-4

**Published:** 2016-10-03

**Authors:** Qian Qin, Shenglin Mei, Qiu Wu, Hanfei Sun, Lewyn Li, Len Taing, Sujun Chen, Fugen Li, Tao Liu, Chongzhi Zang, Han Xu, Yiwen Chen, Clifford A. Meyer, Yong Zhang, Myles Brown, Henry W. Long, X. Shirley Liu

**Affiliations:** 1 Shanghai Key laboratory of tuberculosis, Shanghai Pulmonary Hospital, Shanghai, China; 2 Department of Bioinformatics, School of Life Science and Technology, Tongji University, Shanghai, China; 3 Department of Biostatistics and Computational Biology, Dana-Farber Cancer Institute and Harvard School of Public Health, Boston, MA USA; 4 Center for Functional Cancer Epigenetics, Dana-Farber Cancer Institute, Boston, MA USA; 5 Division of Molecular and Cellular Oncology, Department of Medical Oncology, Dana-Farber Cancer Institute and Department of Medicine, Brigham and Women’s Hospital and Harvard Medical School, Boston, MA USA; 6 Department of Biochemistry, University at Buffalo, Buffalo, NY USA

**Keywords:** ChIP-seq, DNase-seq, Quality atlas, Analysis pipeline

## Abstract

**Background:**

Transcription factor binding, histone modification, and chromatin accessibility studies are important approaches to understanding the biology of gene regulation. ChIP-seq and DNase-seq have become the standard techniques for studying protein-DNA interactions and chromatin accessibility respectively, and comprehensive quality control (QC) and analysis tools are critical to extracting the most value from these assay types. Although many analysis and QC tools have been reported, few combine ChIP-seq and DNase-seq data analysis and quality control in a unified framework with a comprehensive and unbiased reference of data quality metrics.

**Results:**

ChiLin is a computational pipeline that automates the quality control and data analyses of ChIP-seq and DNase-seq data. It is developed using a flexible and modular software framework that can be easily extended and modified. ChiLin is ideal for batch processing of many datasets and is well suited for large collaborative projects involving ChIP-seq and DNase-seq from different designs. ChiLin generates comprehensive quality control reports that include comparisons with historical data derived from over 23,677 public ChIP-seq and DNase-seq samples (11,265 datasets) from eight literature-based classified categories. To the best of our knowledge, this atlas represents the most comprehensive ChIP-seq and DNase-seq related quality metric resource currently available. These historical metrics provide useful heuristic quality references for experiment across all commonly used assay types. Using representative datasets, we demonstrate the versatility of the pipeline by applying it to different assay types of ChIP-seq data. The pipeline software is available open source at https://github.com/cfce/chilin.

**Conclusion:**

ChiLin is a scalable and powerful tool to process large batches of ChIP-seq and DNase-seq datasets. The analysis output and quality metrics have been structured into user-friendly directories and reports. We have successfully compiled 23,677 profiles into a comprehensive quality atlas with fine classification for users.

**Electronic supplementary material:**

The online version of this article (doi:10.1186/s12859-016-1274-4) contains supplementary material, which is available to authorized users.

## Background

ChIP-seq (Chromatin immunoprecipitation followed by high throughput sequencing) is a powerful and widely used technique to map the genome-wide in vivo location of transcription factors, chromatin regulators, and histone modifications. With the growing popularity of this technique and the rapidly dropping cost of next-generation sequencing (NGS), laboratories now are routinely generating more and more ChIP-seq datasets. DNase-seq is a high-throughput technique to map genome wide active cis-regulatory elements based on chromatin accessibility. As of 2016, there are over 20,000 ChIP-seq and DNase-seq samples [1] in the public domain, and this number is increasing rapidly. However, quality control (QC) and analyses of these data have not been straightforward, and the ongoing data explosion poses new challenges and opportunities for the development of computational pipelines for these datasets. First, such computational pipelines must be capable of processing large batches (e.g. ~100-1000s) of data efficiently with minimal user intervention. Second, there is increasing recognition for the importance of ChIP-seq data QC, which should be conducted prior to detailed data analysis and interpretation. Specifically, in the event of a “failed” (low-quality) ChIP-seq or DNase-seq experiment, it will be highly advantageous for a pipeline to explore possible sources of failure that may enable users to modify and improve future experiments. Third, the public availability of tens of thousands of ChIP-seq or DNase-seq datasets represents a rich resource of historical data that can be utilized to facilitate interpretation and identify potential problems.

Although many computational approaches already exist to analyze ChIP-seq data, to the best of our knowledge, there are very few tools that are designed to tackle all three challenges simultaneously. For example, Cistrome [2], CisGenome [3] and ChIPseeqer [4] analysis pipelines provide user-friendly point-and-click solutions that can be conveniently applied only when the number of samples is relatively small. Several new high-throughput computational tools have recently become available for ChIP-seq data analysis, including HiChIP [5], Fish the ChIPs [6], the annotation and visualization modules of Sole-Search [7], seqMINER [8] and the peak calling and motif analysis tool Homer [9]. While powerful, most of these tools focus on ChIP-seq data analysis rather than data QC. ENCODE *phantompeakqualtools* [10], ChIPQC [11], htSeqTools [12], ChIPseeker [13] are R packages specifically designed for ChIP-seq quality control and visualization; however, they all assume users to be familiar with R programming to utilize them. HiChIP implements a ChIP-seq analysis and QC pipeline, but none of its metrics have taken publicly available data into consideration. CHANCE [14], as a standalone QC GUI software and includes ChIP-seq data from ENCODE [15] to help validate experiment quality. However, ENCODE has a limited selection of proteins and histone marks in a limited number of cells compared to all that is available in the public. In addition, ENCODE data quality [16] often represent the best quality ChIP-seq data and thus do not necessarily provide a wide spectrum of data quality against which newly generated data can be compared. Recently, a ChIP-seq QC system and database [17] has collected many useful global and local sample QC indicators for 32,157 publicly available profiles, but none of the indicators account for the ChIP matching input step. Another database integrates limited types of QC metrics for 800 datasets only [18] and, finally, ReMap [19] emphasizes the quality control as well as the downstream analysis of publically available ChIP-seq data, but is focused solely on transcription factors. Therefore, there is an unmet need for a ChIP-seq bioinformatics pipeline that combines data analysis and quality control in a unified framework, with the guidance of a comprehensive data quality atlas.

Here, we present ChiLin, an integrated command line quality control and analysis pipeline for ChIP-seq or DNase-seq data. ChiLin has been exhaustively tested and applied to 11,265 datasets from a wide variety of studies in the public domain obtained via GEO. The atlas of all ChiLin-generated data quality metrics for these datasets is intimately linked to the Cistrome Data Browser (unpublished at http://cistrome.org/db). Further classification of the assay types can divide the QC atlas into assay type independent and dependent QC metrics, which facilitates better understanding of the quality of different assays. ChiLin builds on our previous ChIP-seq and DNase-seq expertise and uses historical data to provide a comprehensive data quality report and analysis results for validating ChIP-seq and DNase-seq experiments.

## Implementation

### Overall design

**Fig. 1:**
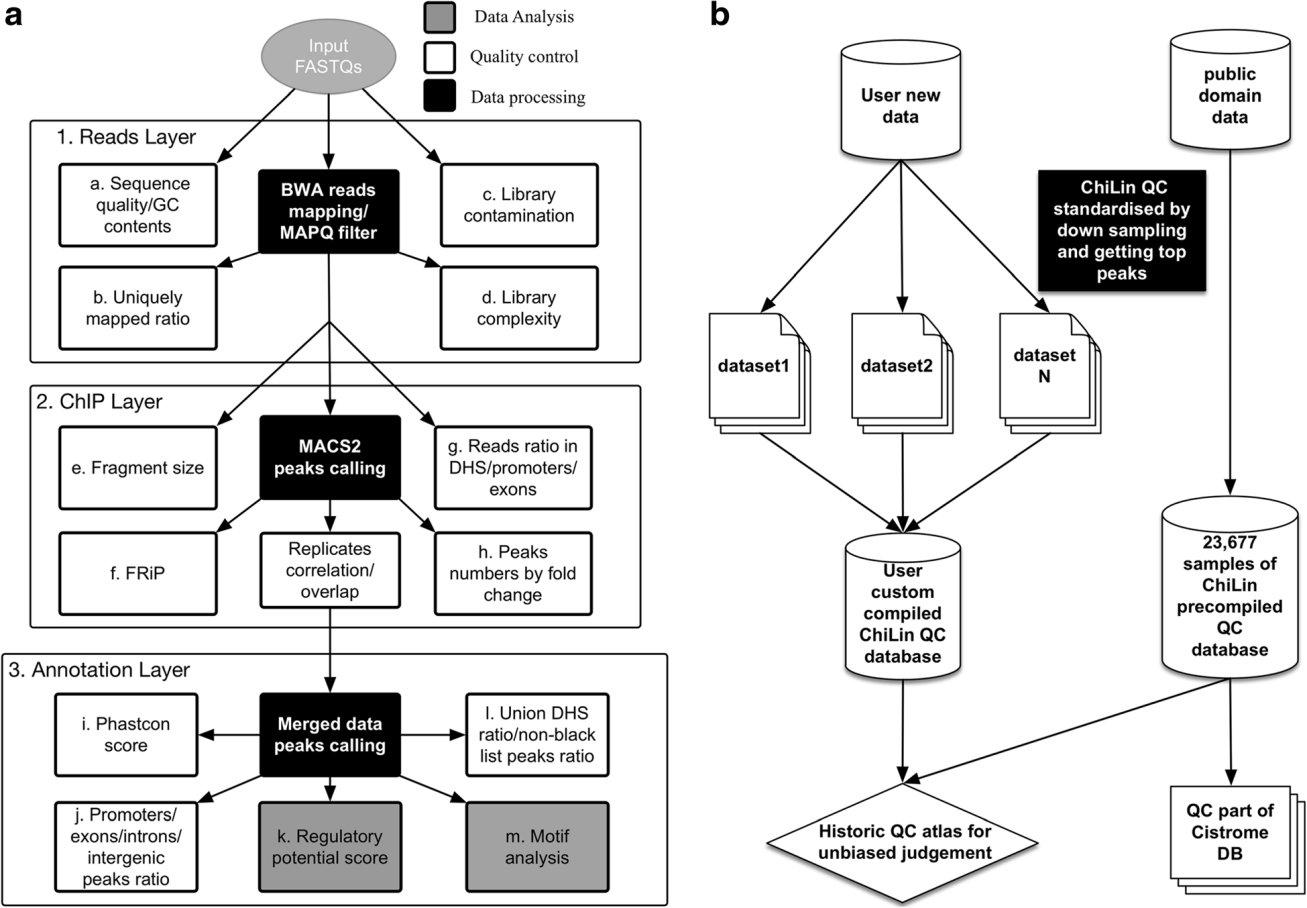
Workflow of ChiLin and quality report. **a** Arrows show the dependency relationship and order of the steps. Data processing steps are indicated in black and quality control steps are indicated in white. **b** batch mode processing of datasets for user to customize internal QC database, post-processing script for compiling QC database is provided at https://github.com/cfce/chilin/blob/master/demo/compile_database.sh

ChiLin is implemented as a python package, and the name represents an omen for luck and prosperity in Chinese tradition. The full documentation is available at http://cistrome.org/chilin. The workflow of ChiLin is illustrated in Fig. 1a, with each box representing a computational step in the ChiLin pipeline. There are data processing steps to perform analyses, and quality control steps to evaluate the results from the analyses. Together, these steps are conceptually grouped into three layers: Read layer, ChIP layer and Annotation layer. Each processing step can be easily extended by other functionality. ChiLin is equipped with a “simple” running mode, where the user specifies all requisite input files and minimal parameters in a single command line; alternatively, ChiLin can read all required inputs parameters from a configuration file, which is a convenient interface for batch processing. The output of ChiLin includes a quality control report in a pdf file, mapped BAM files, a ready-to-view read signal BigWiggle file, narrowPeak and/or broadPeak bed files, for individual replicates and merged replicates, as well as json files containing all quality metrics.

### Read layer

At the Read Layer (Fig. 1a), ChiLin first checks the raw sequence quality and GC content using FastQC [20]. It then maps the ChIP and control FASTQ files onto a user-specified genome build. The default read mapping tool is BWA [21], although the user can also specify Bowtie [22] or STAR [23] for mapping. ChiLin reports the number of reads with mapping quality >1 [24] as “uniquely mapped reads” and the corresponding “uniquely mapped ratio” (uniquely mapped reads over total reads). Beyond the primary mapping target, ChiLin sub-samples 100 K reads from each original library and reports the uniquely mapped ratio for a range of species (at least one of mycoplasma, yeast, human, mouse, and others that can be user-specified) to identify potential sample swaps or contamination.

ChiLin also examines library complexity in terms of unique locations ratio (non-redundant fraction of uniquely mapped reads or NRF) and PCR bottleneck coefficient (PBC) [25] to identify potential over amplification by PCR. “Unique locations” count is the number of genomic locations with one or more uniquely mapped reads. PBC is the number of locations with exactly one uniquely mapped read divided by the number of unique locations. Higher unique location ratio (over all uniquely mapped reads) and higher PBC both indicate sequencing libraries with greater complexity. Since these measures change with sequencing depth, ChiLin calculates these values from a sub-sample of four million uniquely mapped reads so samples with different sequencing depths can be compared (Additional file 1: Figure S1e).

For each user sample, ChiLin reports the percentile of the FastQC score and uniquely mapped ratio compared to the historical data based on a Cistrome ChiLin compiled QC database (Additional file 2: Table S1) of FastQC scores and uniquely mapped ratios from publicly available ChIP-seq samples.

### ChIP layer

The ChIP Layer (Fig. 1a) has quality control metrics that help the user judge the quality of the ChIP enrichment (or quality of the digestion in the case of DNase-seq). ChiLin uses MACS2 (https://github.com/taoliu/MACS/) as the default peak caller. MACS2 estimates fragment size from the cross correlation of reads in the high confidence peaks, and can perform narrow peak (for point source binding) or broad peak (for more diffuse enrichment) calls, or both based on the user specification. In further analysis, ChiLin keeps one unique read for a position for peak calling to reduce false positive peaks. Then it calculates the false discovery rate of the reported peaks by q-value [26], and also reports the fold enrichment of each peak. High quality datasets typically have more peaks, a higher fraction of peaks with >10× enrichment and >20× enrichment, i.e. 10 and 20 fold confident peaks.

ChiLin measures the signal-to-noise ratio of a ChIP-seq data [25] by FRiP, which is the fraction of non-mitochondrial reads in peak regions. Since the FRiP score increases with sequencing depth, ChiLin calculates FRiP from a sub-sampling of 4 M uniquely mapped reads. Another estimate of quality is the percentage of reads that falls in union of DNaseI hypersensitivity sites (DHS). ChiLin derives the union DHS by merging all the peaks of DNase-seq data from ENCODE [27], which represent a comprehensive set of regulatory elements across many cell lines and tissues in the human and mouse genomes.

For datasets with replicates, ChiLin calculates the replicate consistency with two metrics: 1. Pearson correlation of ChIP-seq reads across the genome by using UCSC software wigCorrelate [28] after normalizing signal to reads per million, 2. percentage of overlapping peaks in the ChIP replicates. If more than three replicates available, ChiLin calculates both of the wiggle correlation and peaks overlap count for each pair of the replicates. Then, we divided the peak overlap counts by the larger peak number for each pair of the replicates without considering the difference of the total reads number to deposit into ChiLin quality metrics table (Additional file 2: Table S1). ChiLin merges the raw reads from the replicates and re-runs MACS2 which often yields more robust peak calls.

### Annotation layer

For the Annotation Layer (Fig. 1a) evaluation, ChiLin first plots the average Phastcon conservation profile [29, 30] of all the peaks +/− 2 kb from the peak summits. ChiLin then reports the proportion of peak summits that fall within RefSeq promoter, exon, intron and intergenic regions. ChiLin also reports the percentage of top peaks (default 5000, sorted by MACS score) that fall within union DHS and blacklist regions (“union DHS overlap ratio” and “blacklist overlap ratio”, respectively).

Blacklist regions are a set of regions found by the ENCODE consortium to be consistently enriched in ChIP-seq/DNase-seq/MNase-seq/FAIRE-seq data independent of cell lines and conditions [11, 15, 31]. Union DHS regions, in contrast, represent the whole repertoire of regulatory elements in the human genome. Low blacklist and high union DHS overlap ratios typically indicate good data quality. Phantompeakqualtools [10] requires blacklist regions to unbiasedly compute NSC and RSC score. ChIPQC [11] assess a subsection of its ChIP enrichment quality metrics with blacklist regions filtered out. However, ChiLin computes blacklists overlapping ratio but keeps them in the analysis, since current QC database is built on hg38 and mm10 assembly which lack uniform blacklists. Users can also customize the blacklist regions to compute the overlap ratio for their special needs, such as greyListChIP [32] for cell line specific copy number variation.

ChiLin next performs motif analysis on the top (default 5000) peaks using the Cistrome MDSeqPos [2], a method that weights motifs appearing more frequently at the stronger ChIP-seq peaks and at the center of peaks. Large absolute z-scores of the motifs found from ChIP-seq peaks indicate good motif enrichment and high ChIP-seq data quality. Finally, ChiLin calculates the regulatory potential score, a distance-weighted sum of all the binding sites within 100 KB from the transcription start site, of each RefSeq gene [33]. The list of genes ordered by regulatory potential represents the putative target genes of the protein of interest.

### QC database

We compiled a comprehensive ChiLin quality metrics database of 23,677 public ChIP-seq and DNase-seq samples (11,265 datasets) across the classified eight categories for user reference (Additional file 2: Table S1). We used ChiLin to process public data into datasets, then use post-processing scripts to build the ChiLin QC tables. Users can use ChiLin simple mode to process batches of datasets on their computer clusters, then use the post-processing scripts to compile their custom QC tables (Fig. 1b).

## Results

### ChiLin quality metrics evaluation

**Fig. 2:**
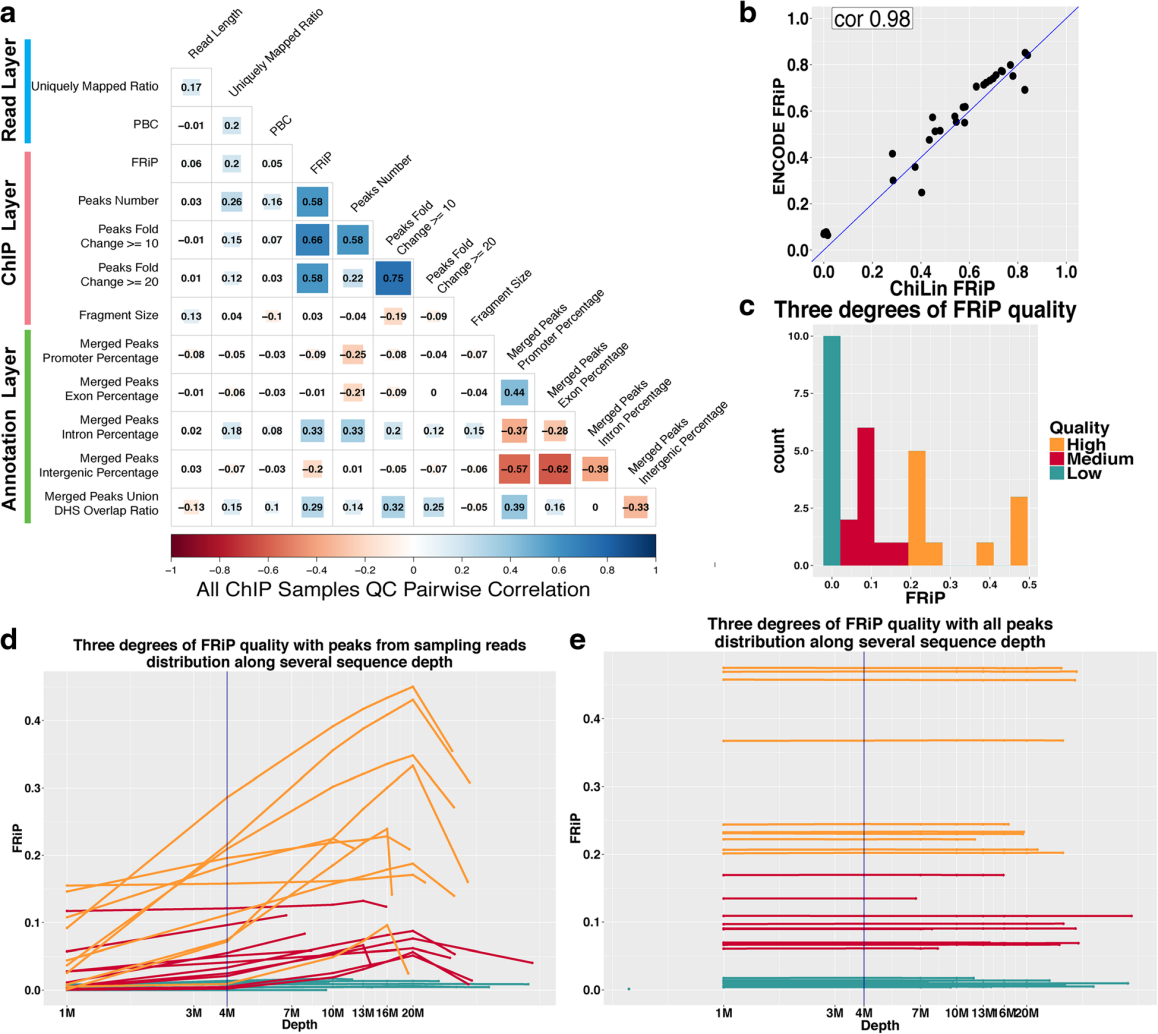
ChiLin Quality metrics exploration. **a** overall ChIP samples quality metrics pairwise correlation across three layers of ChiLin analysis. **b** ENCODE FRiP and ChiLin FRiP score comparison. **c** 30 samples for three degrees of ChIP-layer quality for exploring sequence depth relationship with FRiP. **d** FRiP score distribution along with sequence depth for the samples in c, background is the peak calling with down sampling reads, line and point colours indicates different quality level as in c. **e** FRiP background is the peak calling with all reads

We first evaluated the influence of read length on the stability of ChiLin QC metrics. In general, an increase of read length can result in slight improvements in the uniquely mapping ratio (Fig. 2a, Additional file 3: Figure S3a-d).

We also evaluated all other pairwise relationships across 13 quality metrics. The overall FRiP is positively correlated with peak number across all assay types (Fig. 2a), although peak numbers vary significantly between different assay types from different studies (Fig. 4a). The FRiP scores of chromatin accessibility, transcription factor and chromatin regulators (Additional file 3: Figure S3a,c,d) is highly correlated with peak number, this is not true for histone modification samples (0.37, Additional file 3: Figure S3b) because broad and narrow histone marks are distinctly identified using MACS2 for broad and narrow peak calling mode. In addition, only in DNase-seq is FRiP anti-correlated with estimated fragment size, indicating that shorter fragment length help obtain higher signal-to-noise ratio for DNase-seq (Additional file 3: Figure S3a-d).

Lastly, we evaluated library complexity metric PBC and ChIP enrichment metric FRiP. ChiLin evaluation of PBC and FRiP is highly consistent with ENCODE ones (Fig. 2b, Additional file 1: Figure S1a). The two metrics are variable across studies with different sequence depth. We chose samples based on different ranges of PBC and FRiP to demonstrate this point (Fig. 2c, Additional file 1: Figure S1d). We simulated different sequencing depth by sampling reads from one million up to the total number of reads, and calculated both PBC and FRiP at different sequencing depths. PBC score is largely influenced by sequencing depth (Additional file 1: Figure S1e). For FRiP, two settings are compared: 1. peaks from down-sampled reads, 2. peaks with all reads (overall peaks), which is built in ChiLin. The second setting produced QC statistics that were relatively stable as a function of sequence depth (Fig. 2e), far more so than the first setting (Fig. 2d). Therefore, both of the metrics need to be compared at the same sequencing depth, and FRiP is better calculated by using peaks from all reads. ChiLin evaluates samples with all reads when the number of uniquely mapped reads is less than four million, which may cause potential biases when comparing to other samples (Additional file 1: Figure S1e). More than 93 % samples in this study (22,120 out of 23,677) have more than four million uniquely mapped reads. So, we recommend biologists to produce data with at least four million reads uniquely mapped to the genome for fair quality control.

### Reference data quality atlas with classification

To generate a reference atlas of data quality for ChIP-seq and DNase-seq, we applied ChiLin to all raw sequencing files deposited in Cistrome DB (unpublished), Cistrome CR [34], and CistromeFinder [35]. This analysis resulted in quality metrics for 23,677 samples and 11,265 datasets of ChIP-seq and DNase-seq. In this study, a “sample” corresponds to a single fastq file, whereas a “dataset” has a one-to-one relationship to a unique ChiLin run, which may include multiple “samples” such as replicate IPs and input controls. We summarized the ChiLin-generated quality metrics for all the samples and datasets (Additional file 2: Table S1) used in this study, which are freely available to download and use. The QC database is based on hg38 and mm10, which includes data annotation, classification of assay types (Appendix section Assay Classification), 14 QC metrics across three layers (Fig. 2a), and two replicate consistency metrics (Additional file 2: Table S1). We further divide the 17 QC metrics across three layers into assay type sensitive and insensitive metrics, in order to enable experimental scientists to better associate their own data with ChiLin QC data quality atlas to perform quality control on their sequencing runs, and interpret their data.

**Fig. 3:**
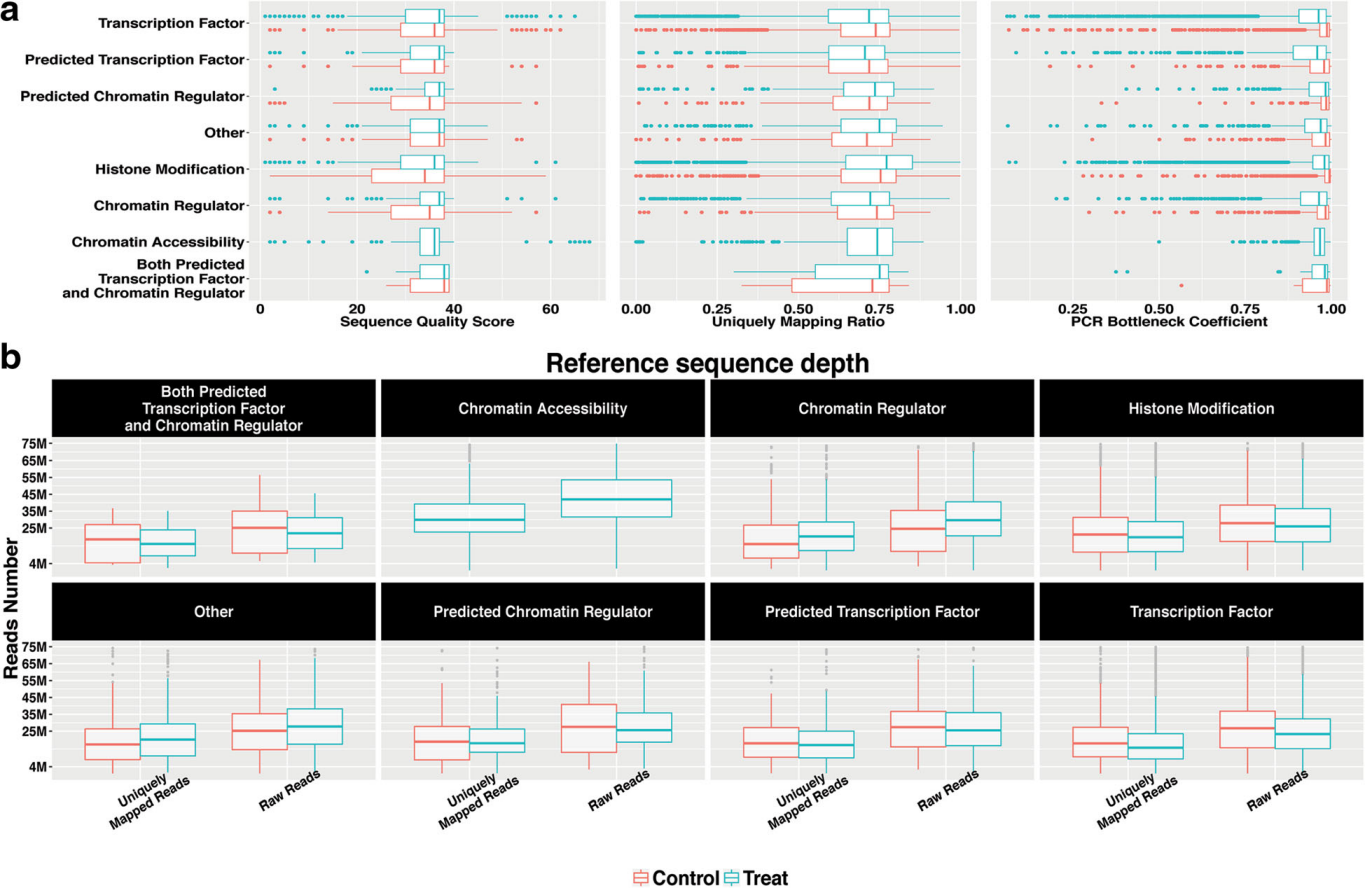
Reads layer quality metrics across eight categories. **a** median sequence quality score from FASTQ files. Uniquely mapped ratio with BWA mapping quality above 1. PCR Bottleneck coefficient calculated from sampling four million reads from BAM files. **b** reference sequence depth suggestions for the eight categories

Combined profiling of sequence quality score, ratio of uniquely mapped reads and PBC can help to establish criteria that will flag potentially low-quality ChIP-seq and DNase-seq data. By looking across assay types, we find no dependence of any of the three metrics on assay types in both ChIP and input samples (Fig. 3a). Thus, the metrics of read layer are assay type insensitive. The cumulative distributions of these metrics (Additional file 4: Figure S4) reveal that >85 % of samples are characterized by sequence quality score >25, uniquely mapped read ratio >0.5 and PBC >0.8. This observation suggests that users may use these cutoffs in assessing the quality of their ChIP-seq data and deciding whether to investigate further. Our QC analysis determined a median sequence depth of 25 million raw reads for ChIP-seq and 40 million for DNase-seq (Fig. 3b).

**Fig. 4:**
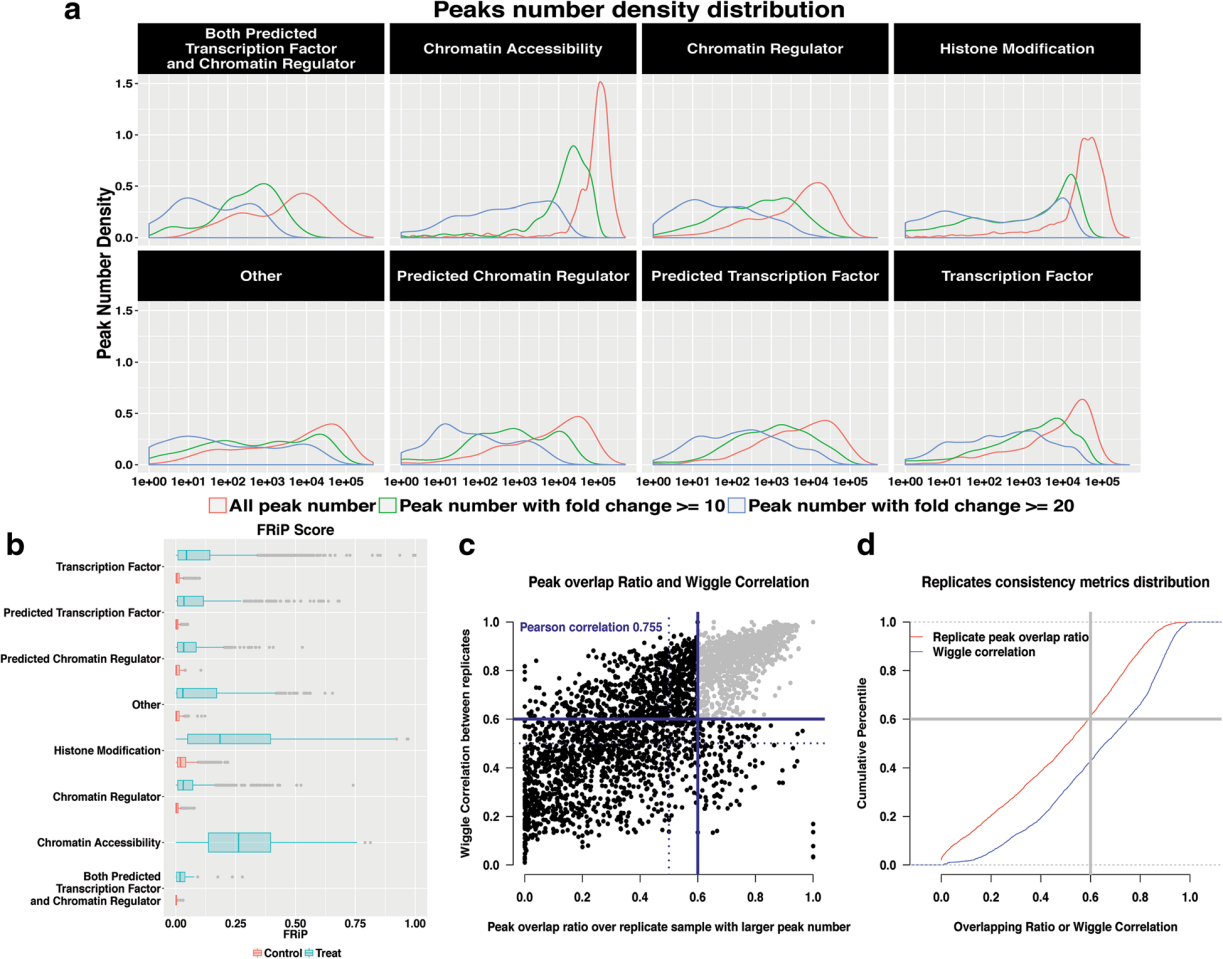
ChIP layer quality metrics across eight categories. **a** MACS2 peak calling of All peaks number, peaks number with fold change >= 20, and >= 10 are displayed density distribution for the eight categories with threshold q value 0.01. **b** Overall FRiP score distributions for ChIP samples (red) and input control samples (cyan) across assay types. **c** Scatterplot of replicates samples wiggle correlation against peaks overlapping ratio. **d** Empirically cumulative distributions of the wiggle correlation and peaks overlapping ratio for the replicates consistency

In contrast to read layer QC, ChIP layer and annotation QC are often sensitive to assay types. Histone marks, chromatin regulators and transcription factors have inherently heterogeneous ChIP enrichment levels, IP samples are typically higher than input control samples for all samples included in our study (Fig. 4b). DNase-seq displays the highest FRiP scores in general (Additional file 5: Figure S5d). Most of the ENCODE datasets have FRiP scores higher than 1 % as well as thousands of peaks as called by MACS2; it is important to note that falling below 1 % FRiP score does not necessarily mean failure [25]. Our observation suggests a FRiP score threshold of 1 % as a proper ChIP enrichment reference for a typical ChIP sample in that the proportion of ChIP samples (80.9 %, 12,032/14,866) much higher than input samples (48.6 %, 4289/8811) (Fig. 4b).

The two quality metrics for replicates consistency correlates well (Fig. 4c). Replicates with sufficient wiggle correlation and peaks overlapping ratio (>0.6, Fig. 4d) are considered to be of high consistency between experiments.

**Fig. 5:**
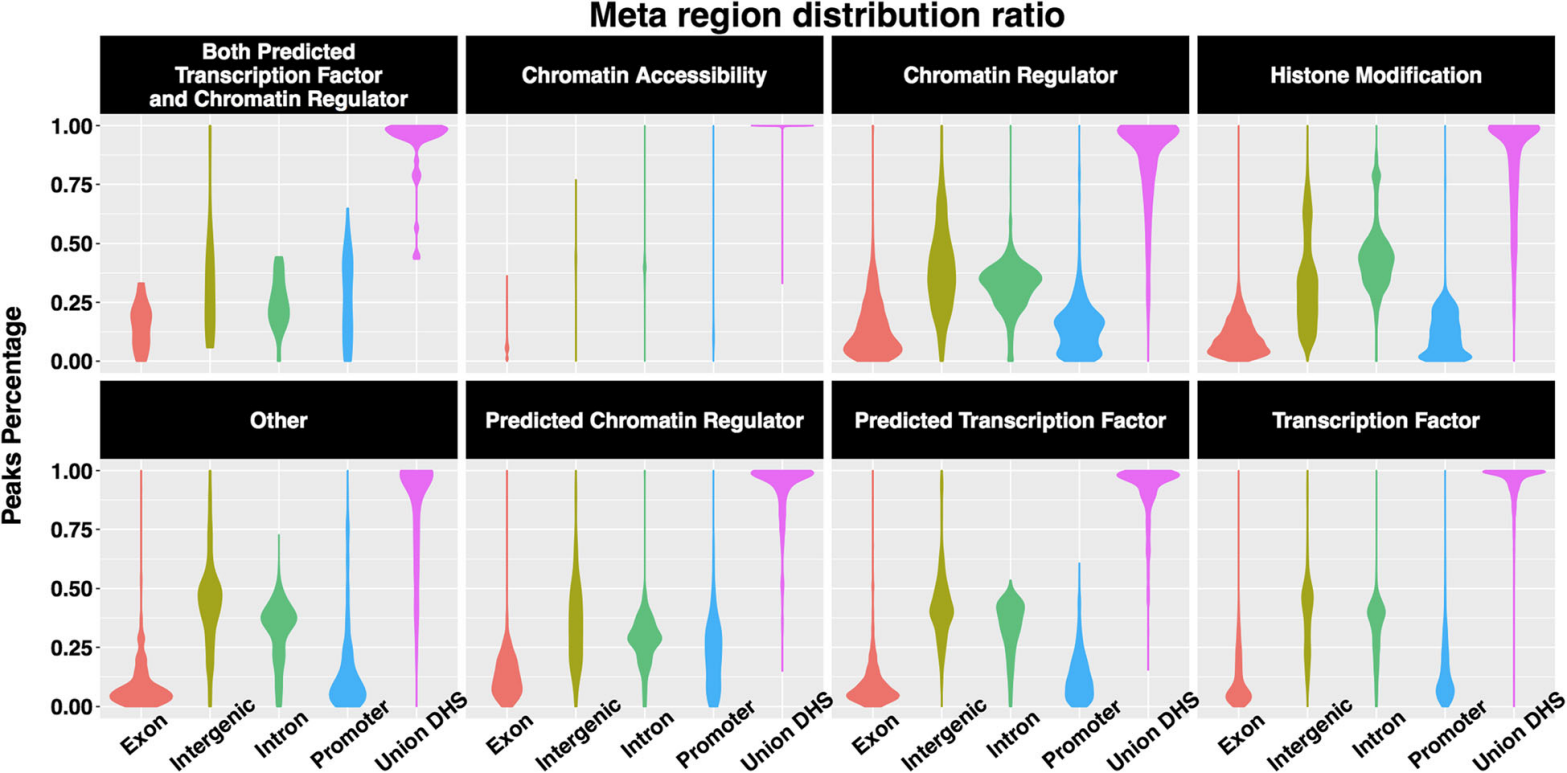
Annotation layer metrics across eight categories. Overall distribution of all peak summit overlapping percentage with exons, introns, promoters and intergenic regions for different categories, and the overall distribution of the ratio of top 5000 peaks overlapping union DHS from ENCODE

Because different assay types are known to possess differential binding preferences for various regions of a genome, experimentalists may find it useful to have access to a comprehensive assessment of the variations in the binding site distributions of different assay types across meta-regions of the genome. Meta regions are defined as genomic regions that had been annotated as promoters, exons, introns, or intergenic regions. We generated meta region metrics distributions across assay types (Fig. 5, Additional file 6: Figure S6) along with the background ratios of the meta regions for human and mouse genome assemblies (Additional file 5: Figure S5, Additional file 7: Figure S7a).

Next, we demonstrate how experimental scientists may use DHS overlap ratio to aid their data interpretation, particularly for histone marks. DHS are commonly identified as regions of accessible chromatin [27], but their exact associations with the diverse set of histone marks have not been fully characterized. Most of chromatin accessibility, transcription factor, and chromatin regulator datasets show a DHS overlap ratio of >0.75. In contrast, histone mark datasets show considerably more variations depending on the specific type of histone mark (Fig. 5, Additional file 6: Figure S6, Additional file 5: Figure S5). We note that most histone acetylation marks (e.g. H3K27ac, H3K122ac, H3K18ac, H4K8ac, H4K91ac, H4K16ac and H4K12ac) show a fairly tight distribution of high overlap with DHS (>0.75), comparable to the transcription factor, chromatin regulator and chromatin accessibility factor types. However, non-acetylation histone marks displays fairly high (e.g. H3T11ph), intermediate (e.g. H3K27me3, H3K36me3), or low (e.g. H3K9me3) values of union DHS overlap ratio (Additional file 5: Figure S5a,b, Additional file 2: Table S1). Since different histone marks play distinct roles in gene regulation, it is noteworthy that many of these histone marks seem to display a broad range of DHS overlap ratios rather than a narrow distribution.

The threshold for each QC metrics is given in the last section of a ChiLin QC report, major metrics are highlighting as blue (good) or red (bad) quality (Additional file 8: File S1–13 Part VII). Users are encouraged to compare their ChIP-seq datasets against corresponding assay types or proteins provided in Additional file 2: Table S1.

### Case studies across factor types

To showcase the utility of ChiLin in performing data QC for a variety of assay types, we present 13 representative examples of actual ChiLin QC reports for both Bowtie and BWA (Additional file 9: Figure S9a-m, Additional file 2: Table S1, Additional file 8: File S1–S13). These reports had been generated from public ChIP-seq datasets of broad histone mark H3K27me3 [36, 37] (Additional file 8: File S1, S2), narrow histone mark H3K4me3 [15, 38] (Additional file 8: File S3, S4), H2A.Z [38] (Additional file 8: File S5), transcription factors TRRAP [39] (Additional file 8: File S6), FOXA1 [40] (Additional file 8: File S7), STAT6 [41] (Additional file 8: File S8), AR [42] (Additional file 8: File S9), the pair end RAD21 [43] (Additional file 8: File S10), and chromatin regulators RAG2 [44] (Additional file 8: File S11) and CHD7 [45] (Additional file 8: File S12), chromatin accessibility DNase-seq [27] (Additional file 8: File S13).

Broad histone mark ChIP-seq are harder to quality control, as indicated by the reference atlas obtained from public data. Neither peak number (Fig. 4a), FRiP score (Fig. 4b), nor DHS overlap ratio (Fig. 5) appears to be a good indicator of data quality for broad marks across different marks. The ChiLin QC reports of H3K27me3 (Additional file 8: File S1, S2) indicate that, although the H3K27me3 datasets meet the basic requirements of read layer quality control, neither of the replicate is of high quality in terms of ChIP layer (e.g. FRiP = 0.04–0.32 %). Nevertheless, the annotation layer flat conservation profiles cannot flag the H3K27me3 quality as inferior since diffuse distribution of H3K27me3 make the broad mark conservation profile spread out in nature. Though, for H3K27me3 in this study, the quality of the dataset with replicates (Additional file 8: File S1) is still better than the one with single sample in terms of larger peaks numbers and FRiP scores (Additional file 8: File S2). For diffuse marks like H3K27me3, ChiLin evaluates FRiP with MACS2 special broad peak calling mode. However, the low FRiP score with broad peaks is still a problem which is not resolved in general, even though there are some alternative tools “*macs2 predictd*” (https://github.com/taoliu/MACS/) for this, whose measure may still not be reliable. Overlapping H3K27me3 with the compiled functional regions, such as HOX genes or EZH2 binding sites is a potential solution, this is still ongoing work for ChiLin.

For the narrow histone marks H3K4me3 and H2A.Z, ChiLin indicates that the datasets used in this study show good overall quality in read and ChIP layers. The H3K4me3 datasets with deeper sequence (Additional file 8: File S3, 19.7 M/20 M) is superior to the one with fewer reads (Additional file 8: File S4, 4.0 M, 4.7 M) in terms of replicates consistency and conservation profile. The lower sequenced datasets of H3K4me3 replicates are not consistent (wiggle correlation 0.02), the 2nd replicate has much lower FRiP and peak number than the 1st one, which may need to be discarded. The higher sequenced H3K4me3 dataset and the H2A.Z dataset is of high quality in terms of high uniquely mapping ratio, PBC, FRiP, peak number, high reproducibility, promoter ratio, union DHS overlap ratio and peaky conservation profile (Additional file 8: File S3, S5).

For transcription factors, chromatin regulators, cohensin and DNase-seq, ChiLin identifies the datasets from AR, FOXA1, CHD7, RAG2, RAD21, and the DNase-seq as being of good quality, and the quality metrics of these datasets are considered to be satisfactory across all the three ChiLin layers (Additional file 8: File S7, S9, S10, S11, S12, S13). Specifically, the motif scan step, which is part of the ChiLin workflow, correctly recovers the corresponding FOXA1, AR, and the cofactor CTCF motifs from AR, FOXA1, and RAD21 ChIP-seq dataset, respectively (Additional file 8: File S7, S9, S10). In contrast, examination of the ChiLin report leads to the conclusion that the TRRAP, STAT6 datasets are likely to be relatively lower quality (Additional file 8: Files S6, S8). Specifically, for STAT6, this is indicated by low FRiP scores (<1 %, which is the average FRiP score for input samples in the reference data quality atlas), relatively low number of peaks (45 in STAT6), poor conservation profiles, and lack of motif enrichment. The TRRAP dataset shows poor wiggle correlation (0.14) and peak overlap count (20) between two biological replicates despite using the same anti-GFP antibody from Abcam, so researchers may need to explore other reasons for the observed low reproducibility, or discard the poor replicate (Additional file 8: File S6).

## Availability and requirements

**Project name**: ChiLin: a comprehensive ChIP-seq quality control and data analysis pipeline

**Project homepage**: http://cistrome.org/chilin/.

**Operating systems**: Linux, MacOS

**Programming language**: Python, R and BASH.

**Other requirements**: bwa (0.7.10), seqtk (1.0), fastqc (0.10.1), samtools (0.1.19), macs2 (2.1.0.20140616), bedGraphToBigWig, wigCorrelate, bx-python (0.7.2), mdseqpos.

**License**: 3-clause BSD.

**Any restrictions to use by non-academics**: follow the license.

## Discussions

Since many excellent tools are already available for ChIP-seq data analysis, we compared ChiLin features with several published computational tools and pipelines (Additional file 2: Table S1). We find that ChiLin is complementary to other tools by providing additional or expanded functionalities. For example, the seqMINER software is a powerful toolset for integrative analysis of multiple ChIP-seq datasets normalization and visualization. The tools CisGenome, HOMER, ChIPseeqer and Sole-Search perform peak calling, peak annotation, motif searches and a series of useful analyses. They currently lack a module dedicated to data quality control. CHANCE is a comprehensive package for ChIP-seq quality control and protocol optimization that compares the user’s data with ENCODE’s large collection of published datasets, but its user-friendly GUI-based design makes batch processing of large collections of data difficult. Similar to ChiLin, HiChIP performs read quality check, read mapping, peak calling and consistency analysis between replicates, data visualization and summary report, and downstream analysis; unlike ChiLin, HiChIP does not report FRiP scores nor utilize historical data for quality metrics, or use known DHS and blacklisted regions from ENCODE in its data quality control analyses. For numerous R-based ChIP-seq analysis tools including CHIPQC, htseqtools, ChIP-seeker and the phantompeakqualtools package used in ENCODE program, quality control is their main concerns and alignment and/or peak files are required as input. Consequently, they can only provide a specific and less comprehensive evaluation based on only a small number of the metrics available in ChiLin. ChiLin has been designed to address all the needs for both data processing and quality control. In combination with the data quality atlas we have assembled, ChiLin is a powerful tool for supporting ChIP-seq and DNase-seq studies of any size.

## Conclusions

ChiLin is an extensible software suite, and it integrates a comprehensive set of QC metrics at various layers of the ChIP-seq and DNase-seq experiments. ChiLin reports these measures in a clear and automatically generated report. ChiLin can process large batches of ChIP-seq and DNase-seq data from single end and paired end experiments. A user can reproduce the quality control and analysis result with only a simple command in a single pipeline process. The incorporation of a simple running mode in ChiLin makes it relatively straightforward to develop customized GUI’s. Therefore, ChiLin can be an attractive solution to rapidly process batches of ChIP-seq datasets in an automated manner with detailed QC reports.

## Appendix

### Assay classification

The purpose of the assay classification is to gain better understanding of the quality metrics since ChIP and annotation layer QC measure is assay category sensitive metrics (Additional file 3: Figure S3a-d, Additional file 6: Figure S6, Additional file 5: Figure S5). We manually grouped the assays into eight categories by literature mining. The literature for classifying assay type is the Cistrome CR [34], histone database HIstome (http://www.actrec.gov.in/histome/) [46], chromatin regulator literature [47, 48], and transcription factor database [49, 50]. First, the assays are scored based on the following rationale (Additional file 2: Table S1): If a factor occurs in one particular reference, the score of the assays increments 0.5 for that category if the reference describes it as a predicted transcription factor or chromatin regulator, otherwise the score of the assays increments 1 for certain transcription factor or chromatin regulator. Then, the scores are summed up to determine the assays’ category following the criteria: 1. If the scores of transcription factor (class a) and chromatin regulator category (class b) are both 0, the assay is assigned to other category. 2. If either of the score of class a or class b is 0.5, then the classification is as below: a. if the score of class a is 0, the score of class b is 0.5, it is a predicted chromatin regulator; b. if the score of class b is 0, the score of class a is 0.5, it is a predicted transcription factor; c. If the score of class a is less or equal than 0.5, the score of class b is larger or equal than 1, it is a chromatin regulator; d. If the score of class b is less or equal than 0.5, and the score of class a is larger or equal than 1, it is a transcription factor; e. If the score of class a equals class b with a score of 0.5, it is assigned to both predicted transcription factor and predicted chromatin regulator. 3. If both of the scores are larger or equal than 1, then (a) if the score of class b is larger than the one of class a, it is chromatin regulator; (b) if the score of class a is larger than the one of class b, it is a transcription factor. The reason for dividing transcription factor and chromatin regulator into predicted, certain or both is to be more accurate and faithful to the literature. For example, TFCat [49] was one of resources we referred to annotate transcription factors, and they classified genes into TF genes, TF gene candidate and so on.

### ChiLin library complexity and aligner effect evaluation

Library complexity of single-end data underestimates the library complexity in comparison with the pair-end ChIP-seq data for Su(Hw) and H3K36me3 [51]. In concept, PBC is more conservative than non redundant tag (NRF) because PBC only consider unique regions with one read against unique regions number instead of considering unique regions against all mapped reads. Further, PBC at lower sequence depth can more accurately estimate library complexity at higher sequence depth in comparison to other methods (https://github.com/matted/census) [52] (Additional file 1: Figure S1b, S1c).

Aligner effects are evaluated in two datasets. For both of the cases, 13 out of 14 metrics are quite similar except to the peaks annotation of union DHS ratio (Additional file 9: Figure S3 a-p, motifs for TRRAP Additional file 8: File S6, FOXA1 Additional file 8: File S7, AR Additional file 8: File S9, RAG2 Additional file 8: File S11, CHD7 Additional file 8: File S12). The union DHS region overlap ratios are variable between aligners because previously we used all peaks to evaluate the union DHS ratio for Bowtie with hg19, and now we used top 5000 peaks for BWA with hg38 (use all peaks if peak number is less than 5000).

### Four categories classification for the most commonly studied assays

Different types of histone modification such as H3K4me3 and H3K27ac indeed have very different characteristics. While it is easy to classify some marks as broad or narrow this is more difficult for others, such as MacroH2A, H2BK120UB, and H2BUB1, that have not been studied as much. We further looked into these well-known broad histone modification (H3K9me3, H3K27me3, H3K36me3), narrow histone modifications (H3K4me3, H3K27ac, H3K4me1), focal contacts (MYC, FOXA1, CTCF for transcription factor, BRD4, EZH2, EP300 for chromatin regulators), and chromatin accessibility (DNase). Broad and narrow histone modifications can be arbitrarily separated by 450 bp of median peaks length (Additional file 5: Figure S5a). FRiP scores for most of the samples excels 1 % suggested thresholds, H3K4me3 and H3K36me3 have higher enrichment for FRiP than other 4 marks. Peaks union DHS sites and promoter regions overlapping ratio can be used as rough guides for separating the active narrow marks from the broad repressive marks (Additional file 5: Figure S5a, b). For the selected focal and chromatin accessibility assays, on average, FRiP scores are higher than the suggested cutoff. FRiP of DNase-seq and CTCF are much higher than the others. The ratio of peak overlap with union DHS sites and promoter for all focal assays and DNase-seq are much higher than the threshold we set for union DHS and the background genomic promoter ratios (Additional file 5: Figure S5c, d). Further, we used H3K9me3 as an example to determine the metric for broad mark by referring to ENCODE ChIP-seq datasets. The overlapping ratio between all public H3K9me3 ChIP-seq broad peak and ENCODE H3K9me3 ChIP-seq union broad peak is larger than 80 % (Additional file 7: Figure S7b).

### ChiLin batch mode and performance

ChiLin support batch processing of datasets in two ways: first for computer servers with limited resources, ChiLin uses the sub-command *batch* to process different configuration files one after another; second, for computer clusters, user can prepare submission scripts with *simple mode*, following the examples publicly available at https://github.com/cfce/chilin/blob/master/demo/. For ChIP treatment of 44.4 million reads with input 34.9 million reads, ChiLin took approximately 9 h 11 min to finish the processing with a single thread, and the output folder was 9.6GB in size. The hardware is CPU- 4x AMD Opteron™ Processor 6378 (64bit, 2.4GHz) each with 16 cores, RAM is 256Gb of DDR3 (1.6GHz). For a batch of 901 datasets, ChiLin took 6 min to run one million reads on average using eight threads on CentOS with Slurm cluster manager software (Additional file 7: Figure S7c).

## Additional files

Additional file 1: Figure S1. Library complexity method exploration. **a.** PBC score comparison between ChiLin and ENCODE. **b.** Correlation between 5 and 30 M uniquely mapped reads across different library complexity metrics for ChIP-seq and DNase-seq. **c.** Linear regresssion F statistics between 5 and 30 M uniquely mapped reads library complexity metrics for ChIP-seq and DNase-seq. **d.** Three degrees of library complexity quality, each with 10 samples. **e.** PBC score distribution at different sequence depths through sampling down the uniquely mapped reads, line and point colors indicates different quality level as in d. (TIF 2123 kb) Additional file 2: Table S1. Sheet1 ToolsComparison: ChIP-seq pipeline software comparison. Sheet2 Examples of ChiLin report. A summary of example data annotation of transcription factor, chromatin regulatory factor and histone modification ChIP-seq data. Sheet3 Protein classification standard for the 8 categories. Sheet4 Protein classification results. Sheet5 BWA QC Database. ChiLin samples and datasets quality metrics across three layers. A clean up table of cistrome samples and datasets quality metrics for ChiLin users’ reference. The QC results is based on the reference of hg38 and mm10 assembly. (XLSX 10363 kb) Additional file 3: Figure S3. Pairwise correlation of ChiLin QC metrics for the four main assay types across read, ChIP and annotation layer. **a.** chromatin accessibility, **b.**histone modification, **c.** transcription factor, **d.** chromatin regulator (TIF 3819 kb) Additional file 4: Figure S4. Cumulative probability fraction of sequence quality score, uniquely mapped ratio, PCR Bottleneck coefficients, MACS2 estimated fragment size across eight categories (TIFF 512 kb) Additional file 5
**Figure S5.** Well-known broad, narrow histone mark, transcription factor, chromatin regulator, and DNase-seq ChIP and annotation layer QC metrics. **a.** narrow histone modification (H3K4me1, H3K27ac, H3K4me3) peaks length distribution along with the FRiP, peaks meta region annotation. Red line for peaks length denotes the 450 arbitrary cutoff to separate broad and narrow mark, red line for FRiP and peaks union DHS overlapping ratio denotes the suggested cutoff of 0.01 and 0.75, red line for meta regions distribution labels the background ratio base pair ratio for exon, intron, promoters and intergenic regions as in Additional file: Figure S7a. **b.** the same as a for broad histone modification (H3K9me3, H3K27me3, H3K36me3). **c.** the same as a for transcription factor (MYC, FOXA1, CTCF) and chromatin regulator (BRD4, EZH2, EP300). **d.** the same as a for all the DNase-seq samples (TIF 2439 kb) Additional file 6
**Figure S6.** Cumulative probability fraction of the ChIP and annotation layer QC metrics (TIFF 918 kb) Additional file 7: Figure S7 a. The reference meta region distribution ratios for four human and mouse assemblies (hg19, hg38, mm9 & mm10). **b.** overall samples H3K9me3 peaks from Cistrome Data Browser overlapping ratio with ENCODE2 H3K9me3 peaks. Constitutive 60, 70, 80 is the genome regions with more than 60 %, 70 %, and 80 % of the ENCODE2 H3K9me3 broad peak locates, union region is the merging genome region of all the ENCODE2 H3K9me3 datasets. **c.** ChiLin runtime performance for 901 datasets. (TIF 1243 kb) Additional file 8: File S1. H3K27me3 data QC report from ChiLin. The sequencing reads had been mapped to the mm10 genome build for BWA and Bowtie. **File S2.** Second H3K27me3 data QC report from ChiLin. The sequencing reads had been mapped to the hg38 genome build for BWA and Bowtie. **File S3.** H3K4me3 data QC report from ChiLin. The sequencing reads had been mapped to the mm10 genome build for BWA and Bowtie. **File S4.** Second H3K4me3 data QC report from ChiLin. The sequencing reads had been mapped to the mm10 genome build for BWA and Bowtie. **File S5.** H2A.Z data QC report from ChiLin. The sequencing reads had been mapped to the mm10 genome build for BWA and Bowtie. **File S6.** TRRAP data QC report from ChiLin. The sequencing reads had been mapped to the hg38 genome build for BWA and Bowtie. **File S7.** FOXA1 data QC report from ChiLin. The sequencing reads had been mapped to the hg38 genome build for BWA and Bowtie. **File S8.** STAT6 data QC report from ChiLin. The sequencing reads had been mapped to the mm10 genome build for BWA and Bowtie. **File S9.** AR data QC report from ChiLin. This report includes two immunoprecipitated and two input samples. The sequencing reads had been mapped to the hg38 genome build for BWA and Bowtie. **File S10.** RAD21 paired end data QC report from ChiLin. The sequencing reads had been mapped to the mm10 genome build for BWA and Bowtie. **File S11.** RAG2 data QC report from ChiLin. The sequencing reads had been mapped to the mm10 genome build for BWA and Bowtie. **File S12.** CHD7 data QC report from ChiLin. The sequencing reads had been mapped to the mm10 genome build for BWA and Bowtie. **File S13.** DNase-seq QC report from ChiLin. The sequencing reads had been mapped to the hg38 genome build for BWA and Bowtie. (GZ 9019 kb) Additional file 9: Figure S9. BWA and Bowtie difference for the ChiLin QC metrics. a-m, 13 QC metrics for the Additional file 8: File S1-S13, datasets resources is described in Additional file 2: Table S1. **n-p.** 230 datasets comparison of hg38 with BWA and hg19 with bowtie (TIF 1028 kb)

## Abbreviations

Cistrome DB: Cistrome Data Browser
DHS: DNase Hypersensitive site
FRiP: Fraction of reads in peaks
IP: Immunoprecipitated
PBC: PCR bottleneck coefficient
QC: Quality control
