# Supplementary figures and images for: ChiLin: a comprehensive ChIP-seq and DNase-seq quality control and analysis pipeline
Source: BMC Bioinformatics. 2016 Oct 3;17:404. doi: 10.1186/s12859-016-1274-4 (PMC5048594; doi:10.1186/s12859-016-1274-4)

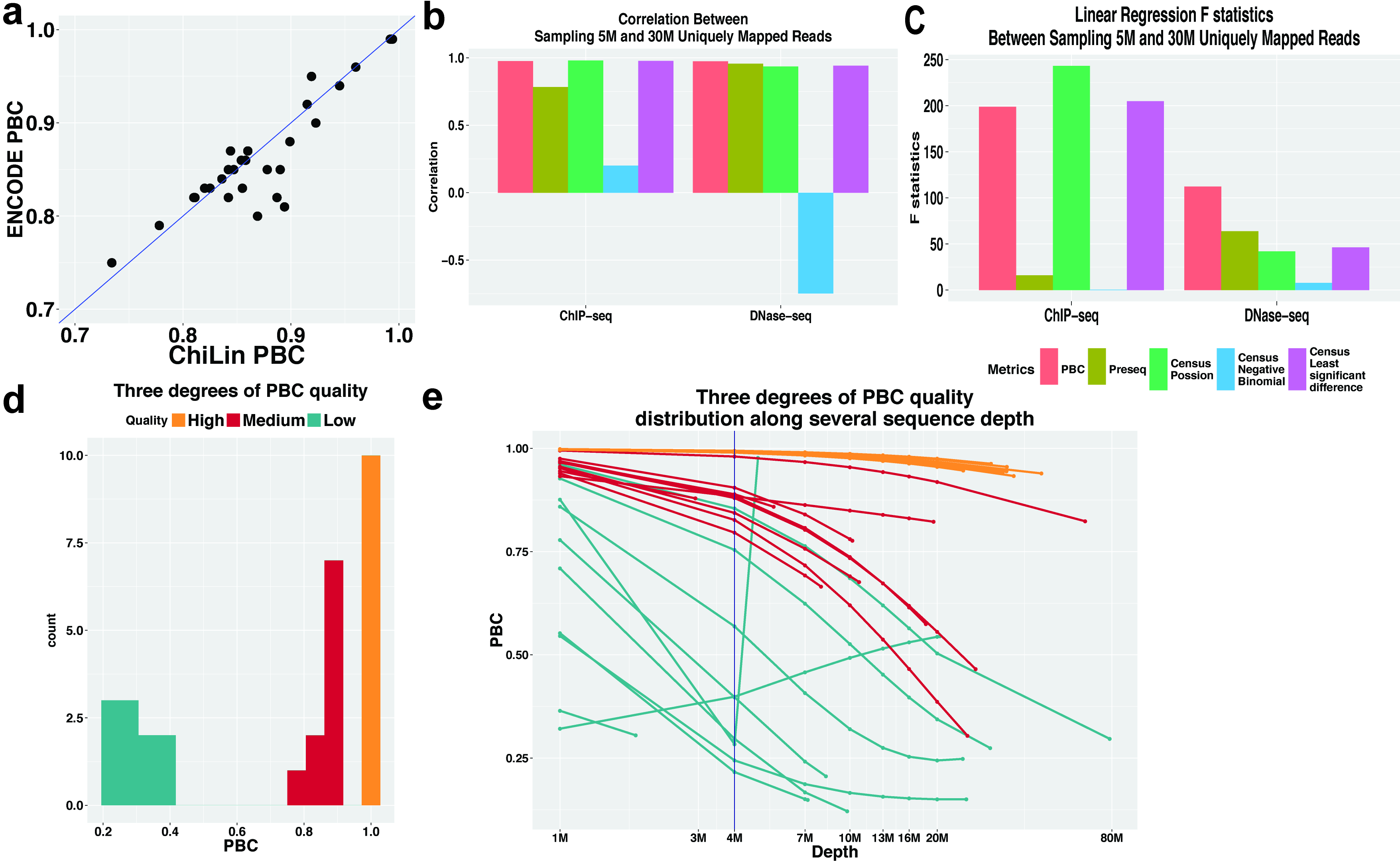

Supplement: Additional file 1: Figure S1. — Library complexity method exploration. a. PBC score comparison between ChiLin and ENCODE. b. Correlation between 5 and 30 M uniquely mapped reads across different library complexity metrics for ChIP-seq and DNase-seq. c. Linear regresssion F statistics between 5 and 30 M uniquely mapped reads library complexity metrics for ChIP-seq and DNase-seq. d. Three degrees of library complexity quality, each with 10 samples. e. PBC score distribution at different sequence depths through sampling down the uniquely mapped reads, line and point colors indicates different quality level as in d. (TIF 2123 kb) [file 12859_2016_1274_MOESM1_ESM.tif]

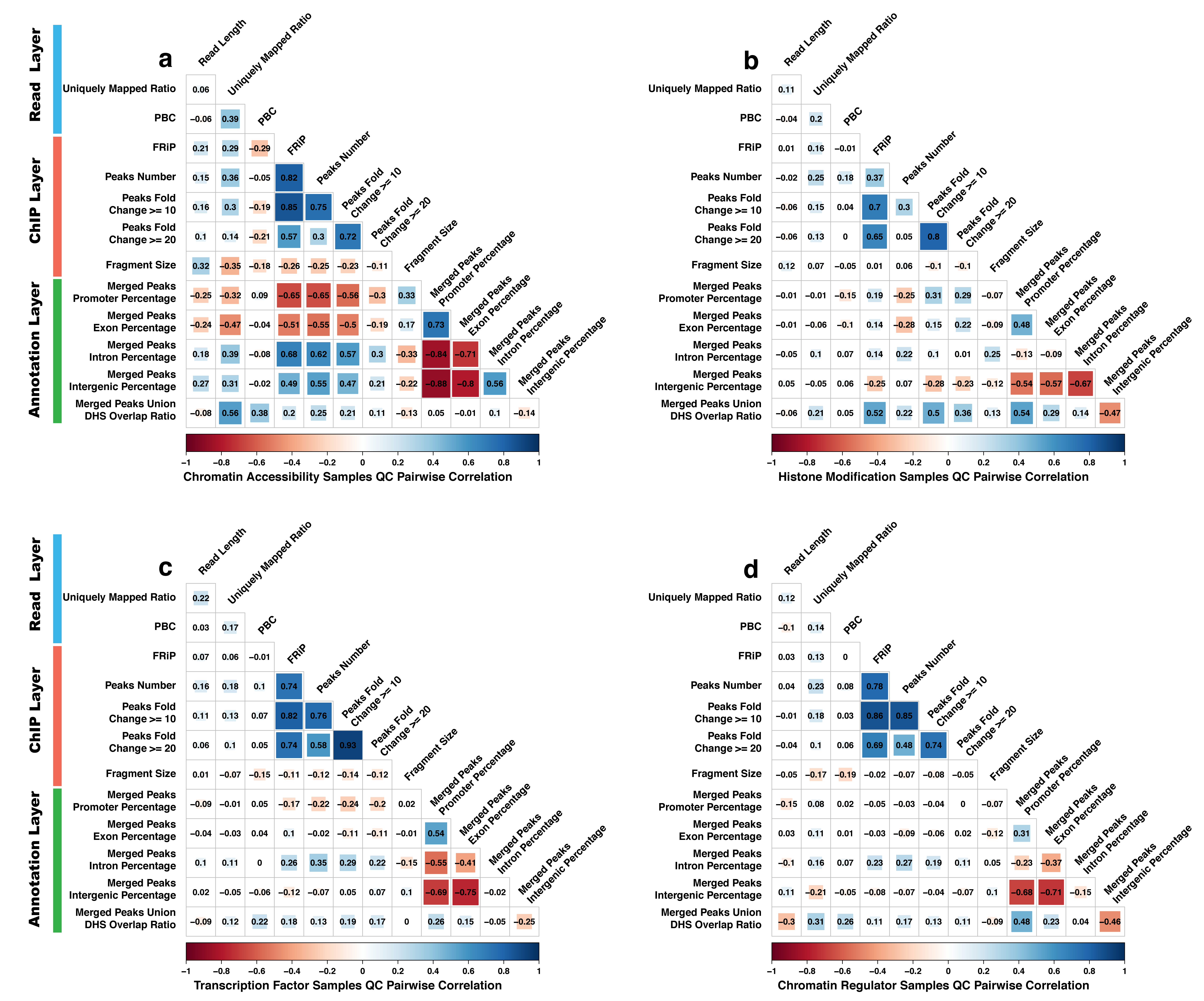

Supplement: Additional file 3: Figure S3. — Pairwise correlation of ChiLin QC metrics for the four main assay types across read, ChIP and annotation layer. a. chromatin accessibility, b.histone modification, c. transcription factor, d. chromatin regulator (TIF 3819 kb) [file 12859_2016_1274_MOESM3_ESM.tif]

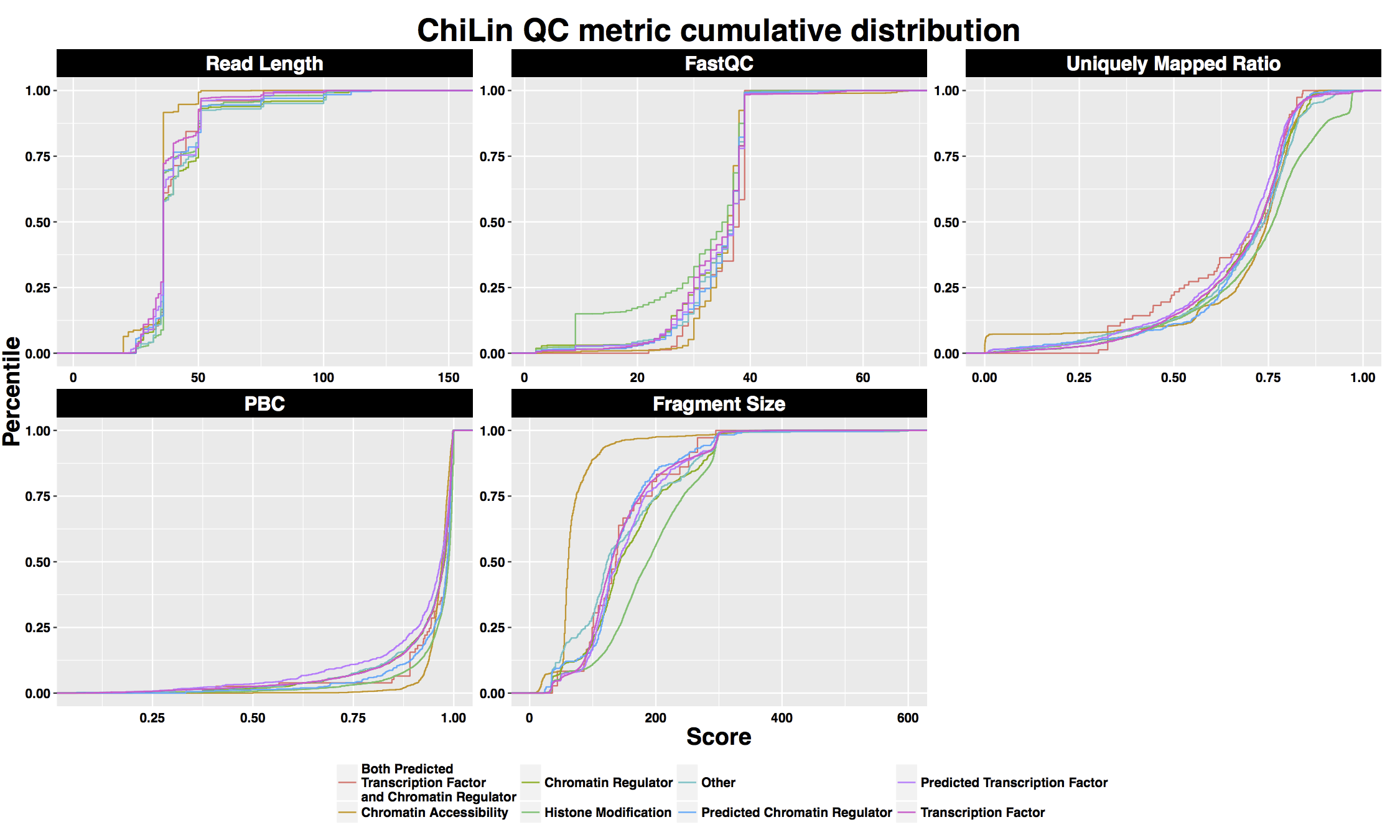

Supplement: Additional file 4: Figure S4. — Cumulative probability fraction of sequence quality score, uniquely mapped ratio, PCR Bottleneck coefficients, MACS2 estimated fragment size across eight categories (TIFF 512 kb) [file 12859_2016_1274_MOESM4_ESM.tiff]

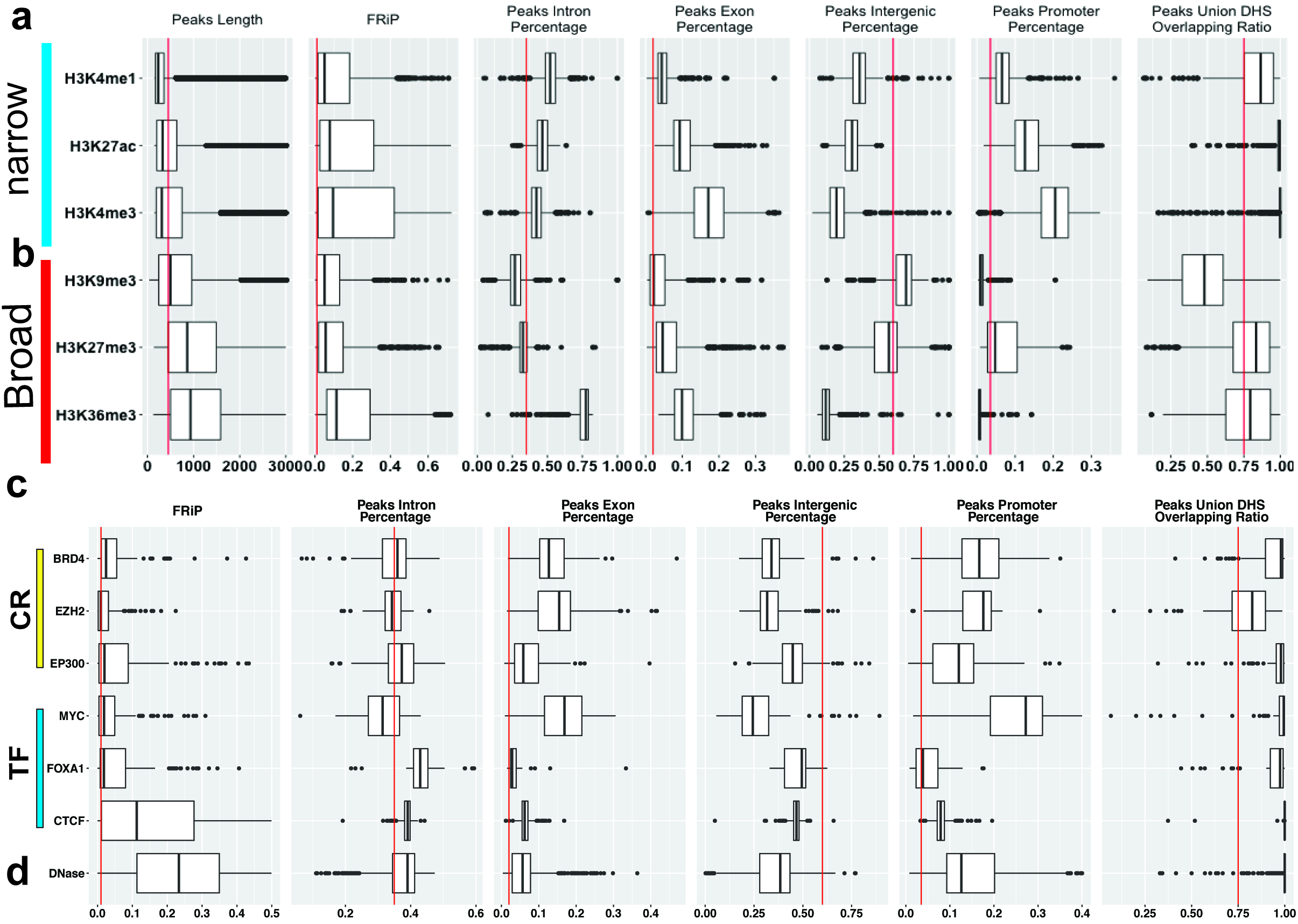

Supplement: Additional file 5 — Figure S5. Well-known broad, narrow histone mark, transcription factor, chromatin regulator, and DNase-seq ChIP and annotation layer QC metrics. a. narrow histone modification (H3K4me1, H3K27ac, H3K4me3) peaks length distribution along with the FRiP, peaks meta region annotation. Red line for peaks length denotes the 450 arbitrary cutoff to separate broad and narrow mark, red line for FRiP and peaks union DHS overlapping ratio denotes the suggested cutoff of 0.01 and 0.75, red line for meta regions distribution labels the background ratio base pair ratio for exon, intron, promoters and intergenic regions as in Additional file: Figure S7a. b. the same as a for broad histone modification (H3K9me3, H3K27me3, H3K36me3). c. the same as a for transcription factor (MYC, FOXA1, CTCF) and chromatin regulator (BRD4, EZH2, EP300). d. the same as a for all the DNase-seq samples (TIF 2439 kb) [file 12859_2016_1274_MOESM5_ESM.tif]

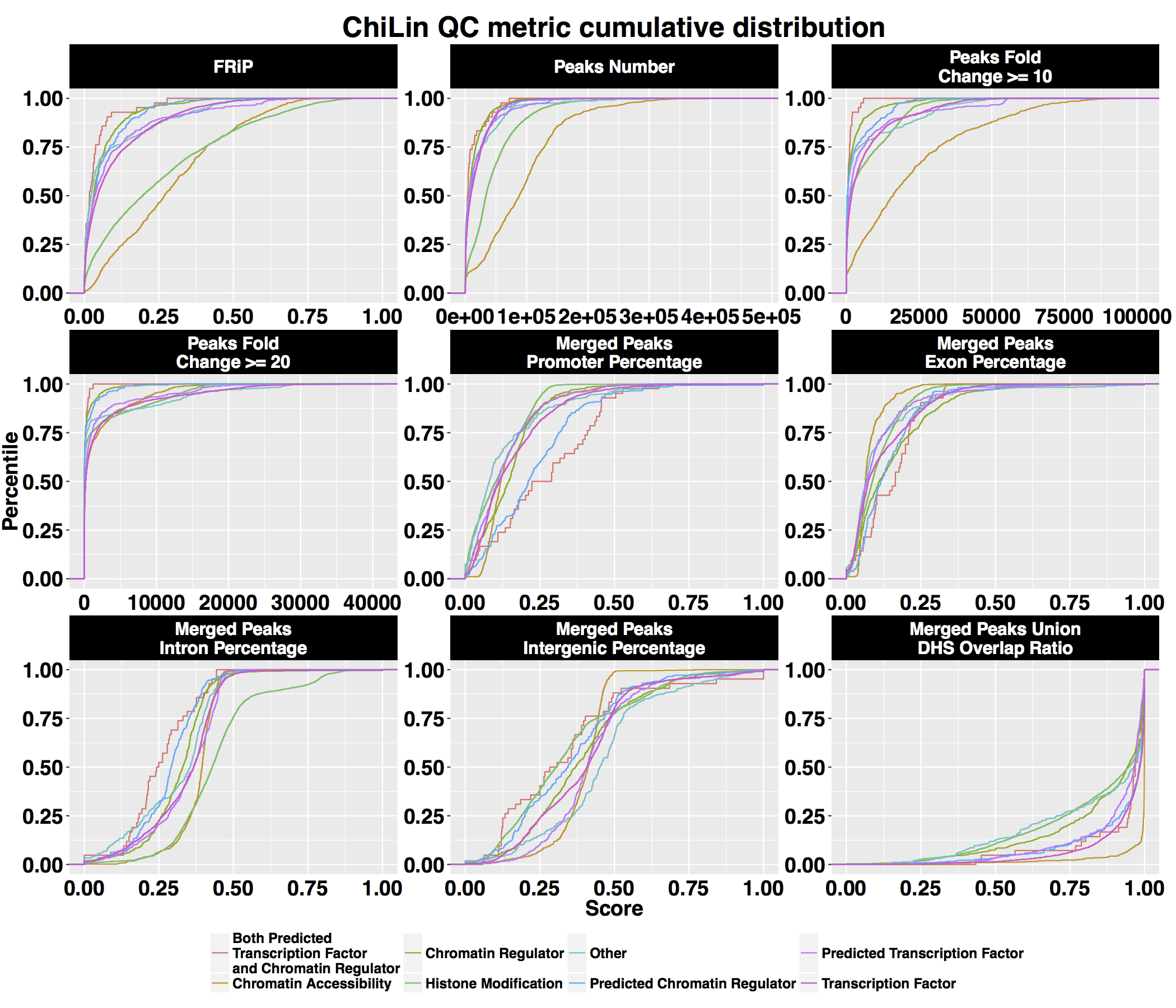

Supplement: Additional file 6 — Figure S6. Cumulative probability fraction of the ChIP and annotation layer QC metrics (TIFF 918 kb) [file 12859_2016_1274_MOESM6_ESM.tiff]

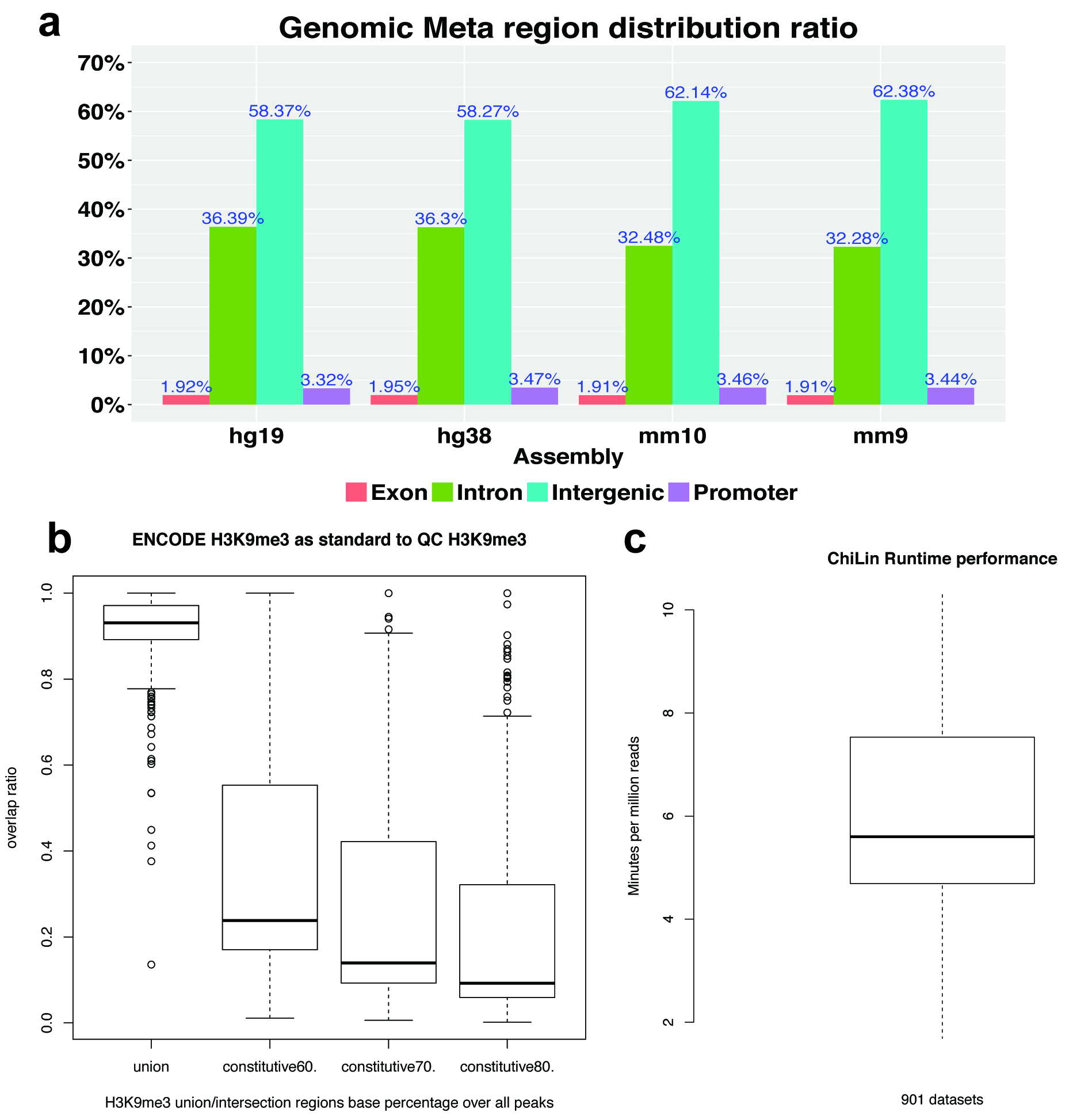

Supplement: Additional file 7: Figure S7 a. — The reference meta region distribution ratios for four human and mouse assemblies (hg19, hg38, mm9 & mm10). b. overall samples H3K9me3 peaks from Cistrome Data Browser overlapping ratio with ENCODE2 H3K9me3 peaks. Constitutive 60, 70, 80 is the genome regions with more than 60 %, 70 %, and 80 % of the ENCODE2 H3K9me3 broad peak locates, union region is the merging genome region of all the ENCODE2 H3K9me3 datasets. c. ChiLin runtime performance for 901 datasets. (TIF 1243 kb) [file 12859_2016_1274_MOESM7_ESM.tif]

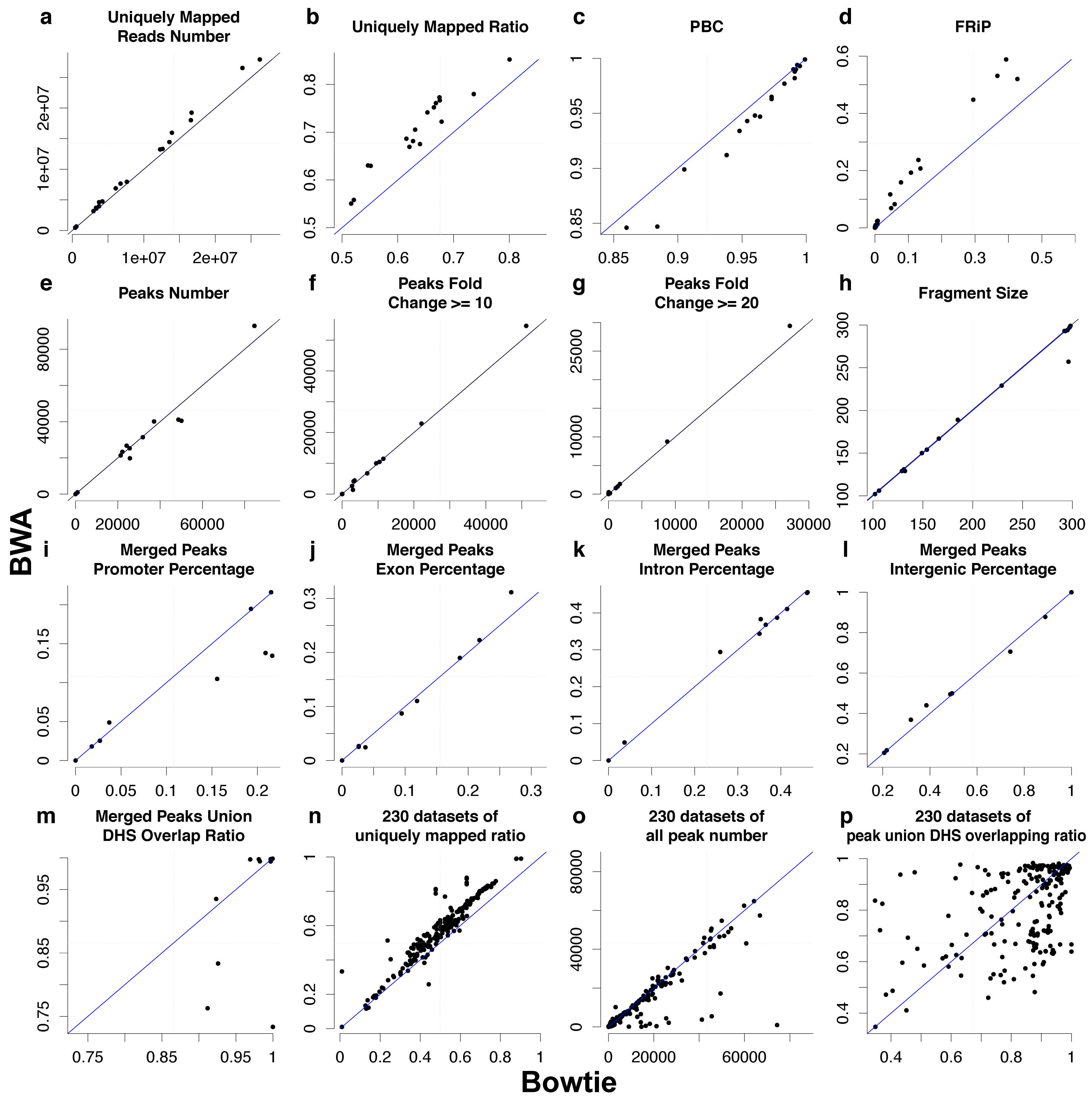

Supplement: Additional file 9: Figure S9. — BWA and Bowtie difference for the ChiLin QC metrics. a-m, 13 QC metrics for the Additional file 8: File S1-S13, datasets resources is described in Additional file 2: Table S1. n-p. 230 datasets comparison of hg38 with BWA and hg19 with bowtie (TIF 1028 kb) [file 12859_2016_1274_MOESM9_ESM.tif]
