# Supplementary material for: ChiLin: a comprehensive ChIP-seq and DNase-seq quality control and analysis pipeline
Source: BMC Bioinformatics. 2016 Oct 3;17:404. doi: 10.1186/s12859-016-1274-4 (PMC5048594; doi:10.1186/s12859-016-1274-4)
Supplement: Additional file 8: File S1. — H3K27me3 data QC report from ChiLin. The sequencing reads had been mapped to the mm10 genome build for BWA and Bowtie. File S2. Second H3K27me3 data QC report from ChiLin. The sequencing reads had been mapped to the hg38 genome build for BWA and Bowtie. File S3. H3K4me3 data QC report from ChiLin. The sequencing reads had been mapped to the mm10 genome build for BWA and Bowtie. File S4. Second H3K4me3 data QC report from ChiLin. The sequencing reads had been mapped to the mm10 genome build for BWA and Bowtie. File S5. H2A.Z data QC report from ChiLin. The sequencing reads had been mapped to the mm10 genome build for BWA and Bowtie. File S6. TRRAP data QC report from ChiLin. The sequencing reads had been mapped to the hg38 genome build for BWA and Bowtie. File S7. FOXA1 data QC report from ChiLin. The sequencing reads had been mapped to the hg38 genome build for BWA and Bowtie. File S8. STAT6 data QC report from ChiLin. The sequencing reads had been mapped to the mm10 genome build for BWA and Bowtie. File S9. AR data QC report from ChiLin. This report includes two immunoprecipitated and two input samples. The sequencing reads had been mapped to the hg38 genome build for BWA and Bowtie. File S10. RAD21 paired end data QC report from ChiLin. The sequencing reads had been mapped to the mm10 genome build for BWA and Bowtie. File S11. RAG2 data QC report from ChiLin. The sequencing reads had been mapped to the mm10 genome build for BWA and Bowtie. File S12. CHD7 data QC report from ChiLin. The sequencing reads had been mapped to the mm10 genome build for BWA and Bowtie. File S13. DNase-seq QC report from ChiLin. The sequencing reads had been mapped to the hg38 genome build for BWA and Bowtie. (GZ 9019 kb) [file 12859_2016_1274_MOESM8_ESM.gz › FileS_bwa/DNase_S13_report.pdf]

CENTER for FUNCTIONAL CANCER EPIGENETICS

ChiP-seq QC Report For

DNase S13

qinq

June 28, 2016

ChiLin Summary

I Summary Table **2**

II FastQC **4**
II.1 FastQC score distribution 5
II.2 FastQC GC content distribution 6

III Basic mapping QC statistics **7**

IV Motif QC analysis **9**

V Library contamination **11**

VI FRiP **13**

VII QC result instruction **15**

Part I

Summary Table

Metrics	treat1	treat2
FastQC	30	30
Original total reads	51.5M	52.0M
Unique mapped reads	37.8M (73.4%)	37.2M (71.47%)
Unique locations of 4M reads	2.5M (63.41%)	2.7M (66.87%)
Locations with only 1 read from 4M reads, number (ratio)	2.4M (59.99%)	2.6M (64.08%)
PBC of 4M reads	94.6%	95.8%
Fragment size of 4M reads	62	63
DHS/Promoter/Exon ratio of 4M reads	70.65%/8.47%/2.15%	61.51%/7.32%/2.07%
FRiP of 4M non-chrM reads	61.29%	47.74%
Replicates total peaks	155759	121140
Replicates 10 fold confident peaks	64396	48263
Replicates 20 fold confident peaks	7194	4068
Replicates reads correlation/replicates peaks overlap	Correlation 0.94/Overlap 116699	
Merged total/10 fold/20 fold peaks	167546/62059/4781	
Top peaks overlap with union DHS number (ratio)	4996 (99.92%)	
Exon/Intron/Intergenic/Promoter ratio of peak summits	4.2%/43.57%/45.35%/6.89%	

Top peaks conservation plot

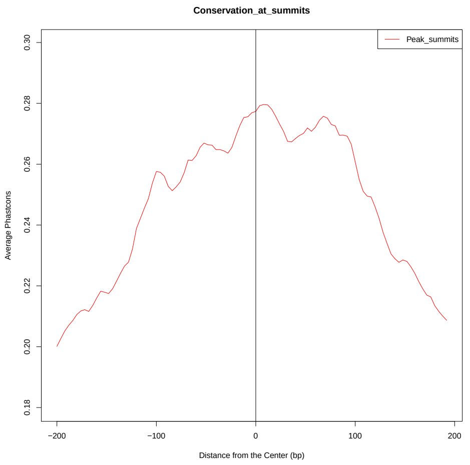

Top peaks motif analysis	JUND -59.5

Part II

FastQC

II.1 FastQC score distribution

We draw the cumulative percentage plot of the FastQC sequence quality scores of all historic data and show how your new data compare(Figure 1).

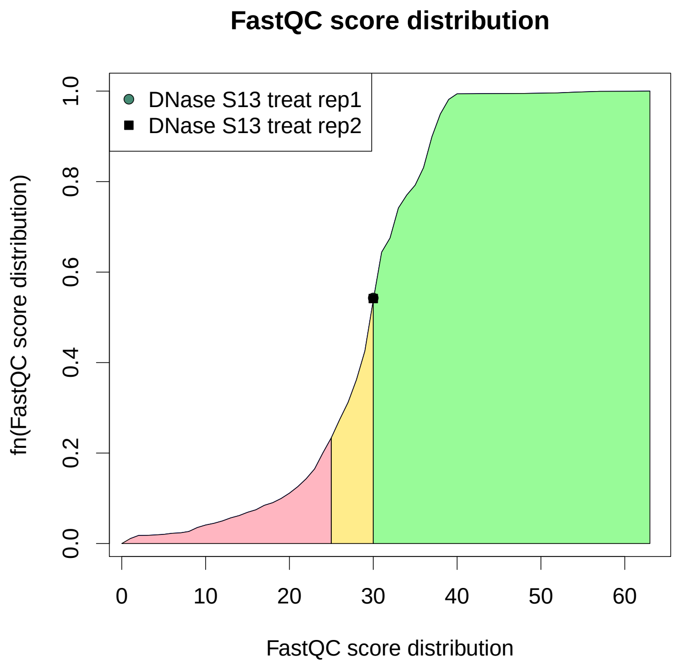

Figure 1: FastQC score distribution plot

II.2 FastQC GC content distribution

We draw the GC content of each of replicates and controls. The expected GC content distribution (i.e. theoretical) is unimodal (Figure: 2). A bi-modal distribution may indicate sample contamination.

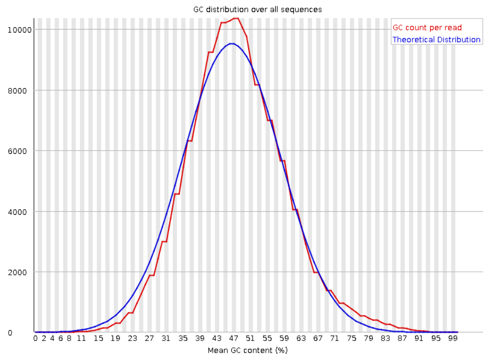

DNase S13 treat rep1

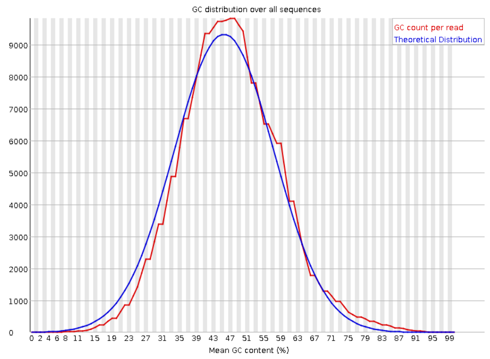

DNase S13 treat rep2

Figure 2: sequence GC content

Part III

Basic mapping QC statistics

We draw the cumulative percentage plot of the mappability rate of all historic data and show how your new data compare.(Figure: 3). The mappability rate is defined by the number of aligned reads divided by the number of total reads. In this plot, the x axis is the mappable rates and the y axis represents the percentage of this mappable rate among all the data. Three color range represent good, medium, and bad quality region.

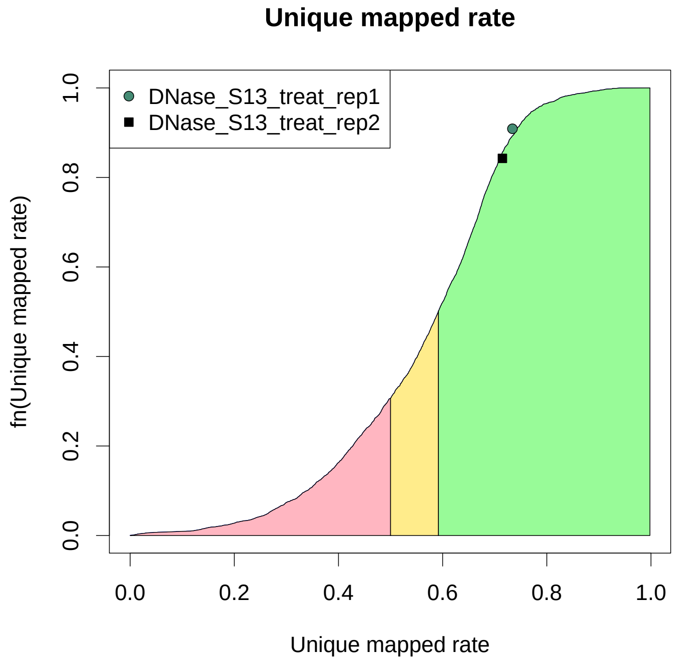

Figure 3: Mappable reads ratio

Part IV

Motif QC analysis

Sequence motifs indicate sequence-specific binding sites for proteins such as nucleases and transcription factors (TF). [1] ChiLin summarize the top motif results (Table 1):

Factor name	Z-score	Hits	Motif logo
*JUND	-59.5209893256	2837	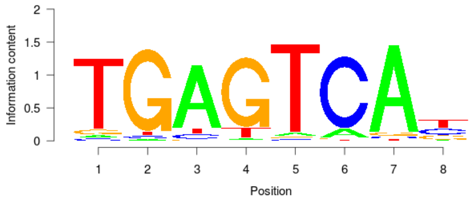
*denovo	-56.7495202983	2790	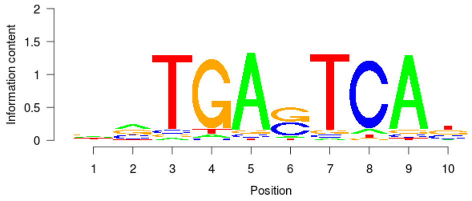
*Jun	-56.4326634778	2726	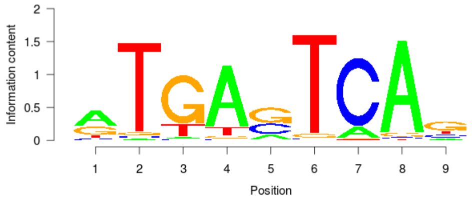
*JUNB	-56.3640699008	2666	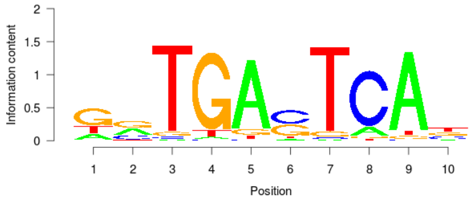
*denovo	-56.3176963575	3082	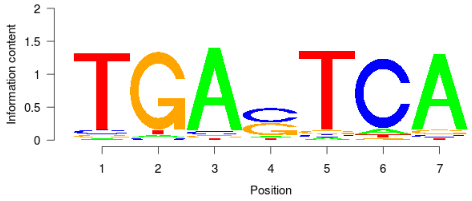

Table 1: Seqpos QC measurement

[1] Patrik D'haeseleer. What are DNA sequence motifs. nature biotechnology 2006,24.

Part V

Library contamination

To check for cross-species contamination of the sample, we randomly select 100K reads from your files, and try to map it to the species defined in the contamination module (in the configuration file). We report the mapping rates (of the random sub-sample) for the various species.(Table 2)

sample name	mycoplasma
DNase S13 treat rep2	0.0%
DNase S13 treat rep1	0.0%

Table 2: Library contamination

Part VI

FRiP

FRiP (Fraction of Reads in Peaks), is a quality measure for ChIP-Seq and Dnase-Seq experiments. FRiP is simply the percentage of all tags that fall in 'hotspots', regions of local enrichment of short-read sequence tags mapped to the genome, i.e. MACS peaks. FRiP scores are positively correlated with true positive rates and replicate reproducibility.

Sample	Reads under Peaks	Total Reads	FRiP
DNase S13 treat rep1	2451641	4000000	61.29%
DNase S13 treat rep2	1909769	4000000	47.74%

Table 3: FRiP measures

Part VII

QC result instruction

1. **FastQC** is the sample's median sequence quality scores. ChiLin calculates these scores using the FastQC software[2]. A good sequence quality score is ≥ 25.
2. **Original total reads** is the sample's raw total reads number.
3. **Uniquely mapped reads** is the number of reads with mapping quality above 1. First, ChiLin aligns reads onto user-specified genomes. Then, it filters the SAM files. The uniquely mapped RATIO is the uniquely mapped reads divided by the total reads. A good uniquely mapped ratio is $\geq 60\%$.
4. **Unique locations of 4M reads** is the number of genomic locations with one or more uniquely mapped reads (unique locations) from sub-sampled 4M reads. Unique locations ratio unique locations number divided by total number of uniquely mapped reads. ChiLin estimates NRF by dividing the number of unique locations by 4M sampled uniquely mapped reads. If reads are less than 4M, then ChiLin uses the total reads instead. ChiLin reports number of unique locations and the unique locations ratio. A good unique locations of 4M reads should be $\geq 70\%$.
5. **Locations with only 1 read from 4M reads number (ratio)** is the number of locations with read number equal to 1 (N1). The ratio is N1 divided by 4M reads unless the total reads is less than 4M, in which case the total reads is used. A good score for this metric is $> 70\%$.
6. **PBC of 4M reads** is N1 (see 5) divided by unique locations (see 4). A good PBC score is $\geq 80\%$.
7. **Fragment size of 4M reads** is in silico estimation of your size selection through maximum cross correlation. The estimation should to be close to the size selected in your experiment.
8. **Exon/DHS/Promoter ratio of 4M reads** is the estimated ratio of reads falling in these regions (from a 4M reads sub-sample). Exons regions are defined as the merged exons regions from the RefSeq gene table. Promoter regions are defined as the RefSeq TSS +/- 2kb regions. Union DHS regions are called from ENCODE II UW DNase-seq Hypersensitive regions. The IP group samples should have higher reads ratios than the control group samples.
9. **FRiP[3] of 4M non-chrM reads** is used for evaluating the signal to noise ratio. First, ChiLin removes chrM reads from the total reads. Then ChiLin sub-samples 4M of these reads. Finally, it calculates the ratio of the sub-sample which fall under the called peaks. A good FRiP score is $\geq 1\%$.
10. **Replicates total peaks** are the total peaks number called by MACS2 with fixed extension size and q value cutoff. A good peaks number depends on your experiment.
11. **Replicates 10 fold confident peaks** are the number of peaks called by MACS2 where the fold change is ≥ 10.
12. **Replicates 20 fold confident peaks** are the number of peaks called by MACS2 where the fold change is ≥ 20.
13. **Replicates reads correlation** is the whole genome reads pearson correlation for all replicates with resolution 146. A good correlation score is ≥ 0.6.
14. **Replicates peaks overlap** is the replicates peaks overlapping number.
15. **Top peaks not overlap with blacklist regions ratio** is the ratio of the merged top 5000 peaks (ordered by MACS2 -log (qvalue)) which do not overlap with blacklist region[4]. This is expected to be $\geq 90\%$.

[2] http://www.bioinformatics.babraham.ac.uk/projects/fastqc/
[3] ChIP-seq guidelines and practices of the ENCODE and modENCODE consortia
[4] https://sites.google.com/site/anshulkundaje/projects/blacklists

16. **Top peaks overlap with union DHS number (ratio)** is the ratio of the merged top 5000 peaks (ordered by MACS2 -log (qvalue)) which overlap with union DHS regions. Union DHS regions are obtained from ENCODE II UW DNase-seq Hypersensitive regions. The union DHS regions was collected from 122 human datasets or 53 mouse datasets, we do not have union DHS of other species. Union DHS generation methods is consisted of three steps: 1.for peaks length longer than 300bp, trim macs2 peaks length to 300bp around macs2 summits, 2.if less than 300bp, preserve the original length, 3.merge the peaks overlap each other. This is expected to be $\geq$ 70%.

17. **Exon/Intron/Integenic/Promoter ratio of peak summits** is calculated using the summits of the merged peaks. ChiLin reports the ratio of overlap with exon, intron, intergenic, and promoter regions for these summits.
We list the background Exon/Intron/Integenic/Promoter ratio here:

Assembly	Exon	Intron	Intergenic	Promoter
hg19	1.92%	36.39%	58.37%	3.32%
hg38	1.95%	36.30%	58.27%	3.47%
mm9	1.91%	32.28%	62.38%	3.44%
mm10	1.91%	32.48%	62.14%	3.46%

18. **Top peaks conservation plot** is the Phastcons conservation scores distribution around +/- 2kb of the top 5000 merged peak summits. Phastcons conservation scores are from placental mammals multiple alignment. For TFs and active histone mark the plot should be shown as a sharp peak in the center.

19. **Top peaks motif analysis** is the motif analysis performed on the top 5000 merged peak summits. These summits are used for discovering highly enriched motifs with MDSeqPos., **Z-score**: a statistical measure of the motif credibility. A good motif will usuaull have a z-score of less than -15. **Hits**: number of times the motif occurs in the top 1000 regions.
